# Lung function in obese children and adolescents without respiratory disease: a systematic review

**DOI:** 10.1186/s12890-020-01306-4

**Published:** 2020-10-28

**Authors:** Mariana Simões Ferreira, Fernando Augusto Lima Marson, Vaneza Lira Waldow Wolf, José Dirceu Ribeiro, Roberto Teixeira Mendes

**Affiliations:** 1 Department of Pediatrics, School of Medical Sciences, Unicamp, Rua Tessália Vieira de Camargo, Cidade Universitária Zeferino Vaz - Barão Geraldo, 126, Campinas, 13083-887 São Paulo Brazil; 2 Department of Pediatrics and Center of Investigation in Pediatrics, Laboratory of Lung Function, School of Medical Sciences, Unicamp, Rua Tessália Vieira de Camargo, Cidade Universitária Zeferino Vaz - Barão Geraldo, 126, Campinas, 13083-887 São Paulo Brazil; 3 Department of Medical Genetics and Genomic Medicine, School of Medical Sciences, Unicamp, Rua Tessália Vieira de Camargo, Cidade Universitária Zeferino Vaz - Barão Geraldo, 126, Campinas, 13083-887 São Paulo Brazil; 4 Postgraduate Program in Health Science, Laboratory of Human and Medical Genetics and Laboratory of Cellular and Molecular Biology and Bioactive Compounds, São Francisco University, Avenida São Francisco de Assis, Jardim São José, 218, Bragança Paulista, 12916-900 São Paulo Brazil

**Keywords:** Lung function, Spirometry, Obesity in adolescence, Obesity in childhood, Review

## Abstract

**Background:**

Obesity in children and adolescents is associated with increased morbidity and mortality due to multisystemic impairment, including deleterious changes in lung function, which are poorly understood.

**Objectives:**

To perform a systematic review to assess lung function in children and adolescents affected by obesity and to verify the presence of pulmonary changes due to obesity in individuals without previous or current respiratory diseases.

**Methods:**

A systematic search was performed in the MEDLINE-PubMed (Medical Literature Analysis and Retrieval System Online), Embase (Excerpta Medica Database) and VHL (Virtual Health Library/Brazil) databases using the terms “Lung Function” and “Pediatric Obesity” and their corresponding synonyms in each database. A period of 10 years was considered, starting in February/2008. After the application of the filters, 33 articles were selected. Using the PICOS strategy, the following information was achieved: (Patient) children and adolescents; (Intervention/exposure) obesity; (Control) healthy children and adolescents; (Outcome) pulmonary function alterations; (Studies) randomized controlled trial, longitudinal studies (prospective and retrospective studies), cross-over studies and cross-sectional studies.

**Results:**

Articles from 18 countries were included. Spirometry was the most widely used tool to assess lung function. There was high variability in lung function values, with a trend towards reduced lung function markers (FEV_1_/FVC, FRC, ERV and RV) in obese children and adolescents.

**Conclusion:**

Lung function, measured by several tools, shows numerous markers with contradictory alterations. Differences concerning the reported results of lung function do not allow us to reach a consensus on lung function changes in children and adolescents with obesity, highlighting the need for more publications on this topic with a standardized methodology.

## Authors summary

### What is known?

(i) Obesity in children and adolescents is a risk factor to higher morbidity and mortality due to multisystemic impairment;

(ii) Obesity as a deleterious factor in lung function is poorly understood.

### What is new?

(i) Spirometry was the most widely used tool to assess lung function in obesity and showed a high variability in its values, with a trend towards reduced lung function markers in children and adolescents with obesity;

(ii) Differences regarding the reported results of lung function do not allow us to reach a consensus on lung function changes in children and adolescents with obesity, highlighting the need for more publications on this topic with a standardized methodology.

## Background

Obesity is a dysfunction that interferes with systems of the body and whose prevalence increases in epidemic proportion [1]. The comprehensive impact of obesity prompts researchers to reflect on its deleterious effects, which progressively worsen the quality of life of increasingly younger individuals, leading children and adolescents to suffer from impairments that had been previously observed in adults only [2, 3].

The respiratory system is one of the systems affected by obesity. Among adults, the most frequent findings in the comparison of lung function of healthy individuals versus individuals affected by obesity are the reduction in functional residual capacity (FRC) and expiratory reserve volume (ERV). One of the main reasons for these changes is the impairment of the respiratory mechanics. Excessive adipose tissue, mainly in the thorax and abdomen, causes an increase in the intra-abdominal pressure on the diaphragm and in the pressure of the fat tissue on the rib cage, hindering thoracic expansion and, consequently, lung compliance. This change leads to a reduction in lung volumes and capacities and is characteristic of a restrictive lung disease [4, 5].

Respiratory mechanics are not the only way of compromising lung function in obesity. There is also an inflammatory component that causes obstructive pulmonary disorders. Adipose tissue macrophages produce proinflammatory substances and adipocytes secrete hormones (adipokines), which reach the systemic circulation, and are able to act directly on the respiratory system or alter the immune response. The entire pathophysiological process favors the induction of bronchial hyperreactivity and may compromise pulmonary air flow [6–8].

The prevalence of obesity has increased worldwide, and although it is a relevant public health problem that affects all age groups, the role and methods to evaluate its impact on lung function in children and adolescents is still unclear, and full understanding of the topic is still far from being attained.

**Fig. 1:**
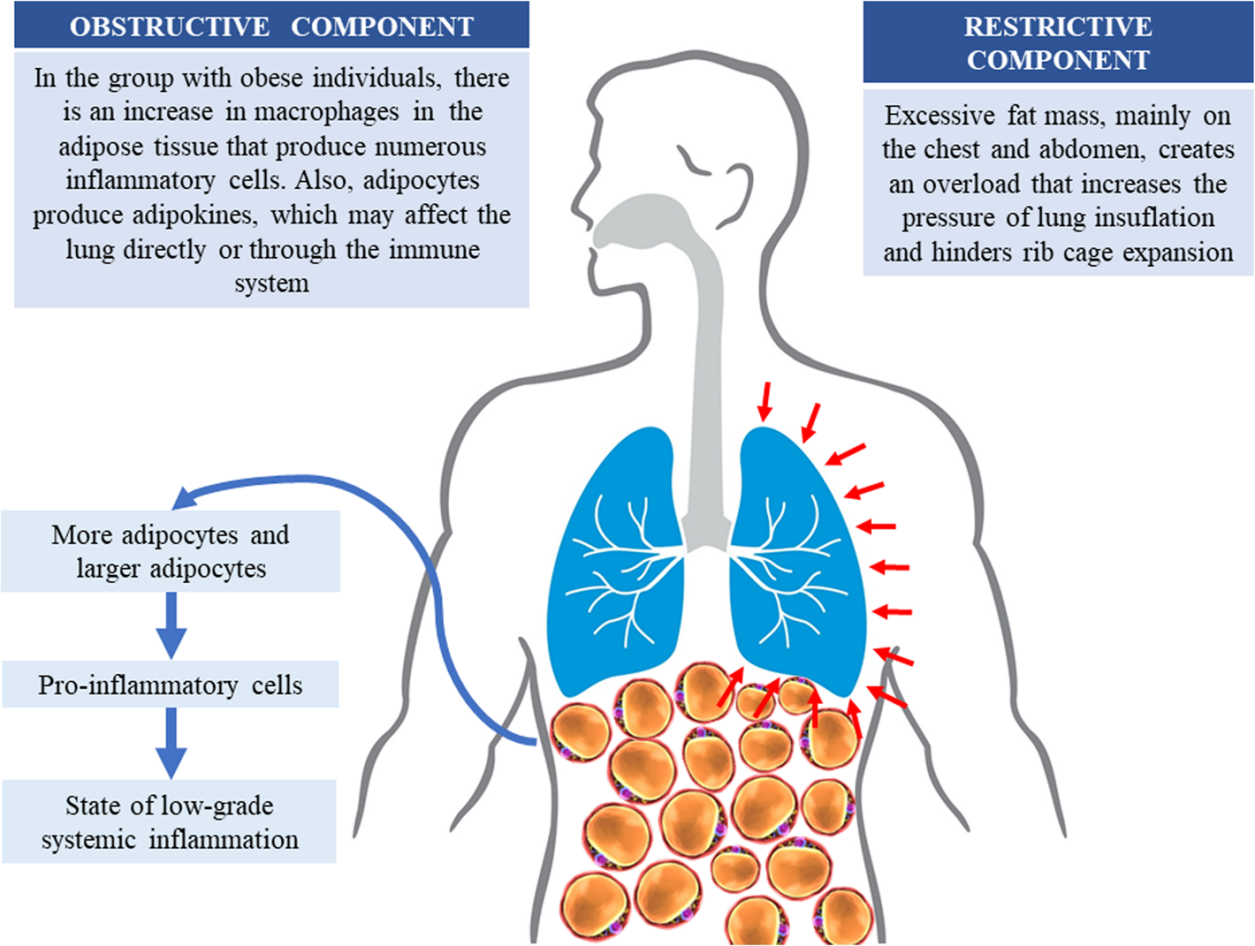
Mechanical and obstructive adipocytes related components that influence lung function in children and adolescents with obesity. The images used in the figure are not under copyright

As the obesity and lung function are complex phenotypes and their interaction has not been well understood, we included Fig. 1 that summarizes the mechanisms of lung function impairment due to obesity. Despite the relevance of the topic, it is still not well-established when lung function damages related to obesity start. Studies with children and adolescents diverge in their conclusions. Body changes during childhood and adolescence, variations in age range as well as in ethnic/environmental/genetic specificities make understanding of the cause-effect relationship between obesity and lung function more difficult.

Obesity and its association with lung function have been more often studied to ascertain the diagnosis of patients with asthma. Obesity and asthma have shown increasing prevalence in the last decades, and at the same time, they share common aspects, including the inflammatory process [8–10].

Obesity and asthma have been described as concomitant risk factors, with characteristics of a cause-effect relationship. The assessment of lung function in individuals with obesity and without asthma has yielded mixed results. Comprehensive studies are needed to understand whether mechanical and inflammatory changes are present in childhood obesity and during the growth process. Further studies should verify whether the disorders manifest differently during childhood and adolescence due to body changes throughout these periods, especially in individuals without a known lung disease, which is the focus of this systematic review [8–10].

This systematic review aimed to assess lung function in children and adolescents with obesity and to verify the presence of pulmonary restrictive or obstructive damages due to obesity in individuals without previous or current respiratory diseases, including asthma.

## Methods

A systematic search was conducted in MEDLINE-PubMed (Medical Literature Analysis and Retrieval System Online, Public Medline), Embase (Excerpta Medica Database) and VHL (Virtual Health Library – Brazil) databases. Platform-specific tools were used, considering the descriptors (PubMed – MeSH Terms, VHL – DeCS and Embase – Emtree Terms) and equivalent terms, as well as excluding the descriptor “asthma” in order to achieve the objective of the study. The terms used in each database are described in Table 1.

**Table 1:** Descriptors used for the database search MEDLINE-PubMed (Medical Literature Analysis and Retrieval System Online-Public Medline), Embase (Excerpta Medica Database) and VHL (Virtual Health Library-Brazil)

Term	PubMed (MeSH Terms)	VHL (DeCS)	Embase (Emtree Terms)
Pediatric obesity	Childhood obesity	Childhood obesity	Childhood obesity
	Adolescent obesity	Obesity, pediatric	Child obesity
		Obesity, childhood	Obesity in adolescence
		Childhood onset obesity	
		Obesity, childhood onset	
		Child obesity	
		Obesity, child	
		Childhood overweight	
		Overweight, childhood	
		Obesity in childhood	
		Adolescent obesity	
		Obesity, adolescent	
		Obesity in adolescence	
		Adolescent overweight	
		Overweight, adolescent	
Lung function	Respiratory function tests	Function tests, pulmonary	Respiratory function
	Spirometry	Function test, pulmonary	Lung function test
		Pulmonary function test	Spirometry
		Test, pulmonary function	
		Tests, pulmonary function	
		Function test, lung	
		Function test, respiratory	
		Function tests, lung	
		Function tests, respiratory	
		Lung function test	
		Respiratory function test	
		Tests, respiratory function	
		Lung function tests	
		Pulmonary function tests	

**Fig. 2:**
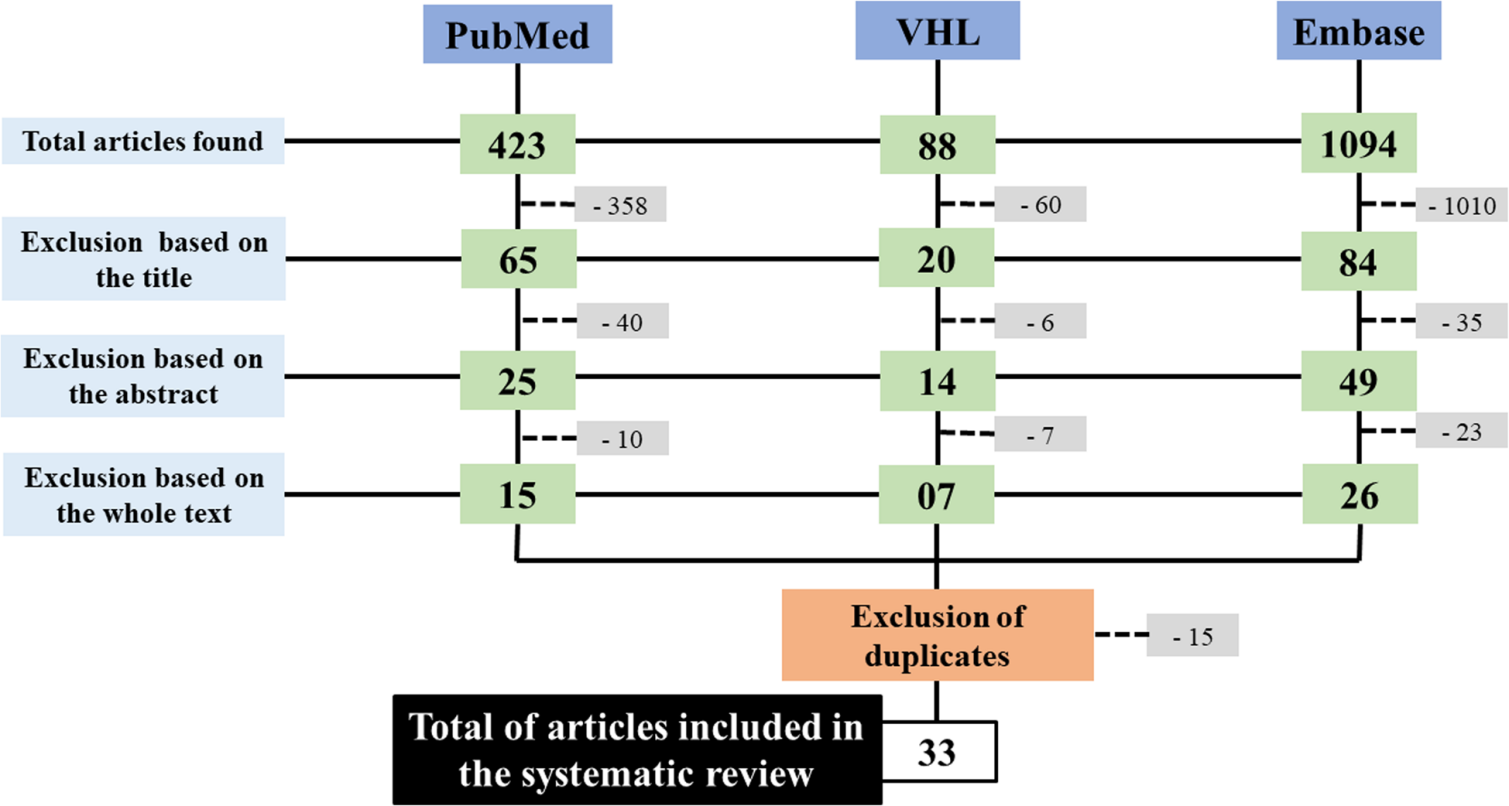
Flowchart with distribution of articles according to the databases and selection filters used

The articles were selected in three stages, as detailed in Fig. 2. The titles were first read and the articles that were not relevant to the review were excluded (358, 1,010 and 68 articles found in PubMed, Embase and VHL, respectively, were excluded). After the first database search filter was used, the articles were selected based on the abstracts (40, 35 and six articles found in PubMed, Embase and VHL, respectively, were excluded). In the third stage, the articles were read in full and then, carefully screened according to their relevance to the topic (10, 23 and seven articles found in PubMed, Embase and VHL, respectively, were excluded). Also, using the PICOS strategy, the following information was achieved: Patient – children and adolescents; Intervention (Exposure) – obesity; Control – healthy children and adolescents; Outcome – pulmonary function variability; Studies – randomized controlled trial, longitudinal studies (prospective and retrospective studies), cross-over studies and cross-sectional studies.

### Eligibility criteria

In our study we included the following types of studies: randomized controlled trial, longitudinal studies (prospective and retrospective studies), cross-over studies and cross-sectional studies. Also, there was no restriction regarding the length of follow-up, and we considered only studies published between 2008 and 2018. We considered studies published in English, Spanish or Portuguese, and that were available in full text.

### Information sources

All the studies were collected from the following databases: MEDLINE-PubMed, Embase and VHL databases. All the data was extracted directly from the studies and there was no contact with the study authors.

### Search in the databases using descriptors

The following strategies were used to perform all searches in the study:

MEDLINE-PubMed.

(((((“Lung function”[Title/Abstract]) OR ((Respiratory Function Tests [MeSH Terms]) OR “Respiratory Function Tests”[Title/Abstract])) OR ((Spirometry [MeSH Terms]) OR Spirometry[Title/Abstract]))) AND (((((Obesity[MeSH Terms]) OR Obesity[Title/Abstract])) OR ((Pediatric Obesity[MeSH Terms]) OR “Pediatric Obesity”[Title/Abstract])) OR ((((((Childhood Obesity[MeSH Terms]) OR “Childhood Obesity”[Title/Abstract]) OR Adolescent Overweight[MeSH Terms]) OR “Adolescent Overweight”[Title/Abstract]) OR Adolescent Obesity[MeSH Terms]) OR “Adolescent Obesity”[Title/Abstract]))) NOT ((Asthma[MeSH Terms]) OR Asthma[Title/Abstract])

VHL

((tw:(Pediatric Obesity)) OR (tw:(Obesity, Pediatric)) OR (tw:(Childhood Obesity)) OR (tw:(Obesity, Childhood)) OR (tw:(Childhood Onset Obesity)) OR (tw:(Obesity, Childhood Onset)) OR (tw:(Child Obesity)) OR (tw:(Obesity, Child)) OR (tw:(Childhood Overweight)) OR (tw:(Childhood Overweights)) OR (tw:(Overweight, Childhood)) OR (tw:(Obesity in Childhood)) OR (tw:(Infant Obesity)) OR (tw:(Obesity, Infant)) OR (tw:(Infant Overweight)) OR (tw:(Overweight, Infant)) OR (tw:(Infantile Obesity)) OR (tw:(Obesity, Infantile)) OR (tw:(Adolescent Obesity)) OR (tw:(Obesity, Adolescent)) OR (tw:(Obesity in Adolescence)) OR (tw:(Adolescent Overweight)) OR (tw:(Overweight, Adolescent))) AND ((tw:(Lung function)) OR (tw:(Function Tests, Pulmonary)) OR (tw:(Function Test, Pulmonary)) OR (tw:(Pulmonary Function Test)) OR (tw:(Test, Pulmonary Function)) OR (tw:(Tests, Pulmonary Function)) OR (tw:(Function Test, Lung)) OR (tw:(Function Test, Respiratory)) OR (tw:(Function Tests, Lung)) OR (tw:(Function Tests, Respiratory)) OR (tw:(Lung Function Test)) OR (tw:(Respiratory Function Test)) OR (tw:(Test, Lung Function)) OR (tw:(Test, Respiratory Function)) OR (tw:(Tests, Lung Function)) OR (tw:(Tests, Respiratory Function)) OR (tw:(Lung Function Tests)) OR (tw:(Pulmonary Function Tests))) AND NOT (asthma)

### Study selection

In brief, the study selection was carried out as represented in Fig. 2. Also, two authors (MFS and FALM or MFS and VLWW) decided about the eligibility before including the study in the review. In the presence of ambiguous conclusion, a third author (RTM or JDR) was contacted to perform a full consideration. Afterwards, a fourth author revised all the studies and the dataset to reach a final decision (RTM or JDR).

### Data collection process

The data collection was carried out by two authors (MFS and FALM or MFS and VLWW), in this way, the data collection was performed twice for each study. Also, after the data extraction, the study was described as Table 2 and both authors included a summary using both datasets generated in the individual data collection. In the presence of ambiguous information, a third author (RTM or JDR) was contacted to perform a full consideration.

**Table 2:** Descriptive analysis of the articles included in the systematic review

Authors (years) - country	Objective	Exclusion of respiratory diseases?	Type of study	Population	Lung function	Standardized instrument and position for assessment	Marker of lung function (Intervention)	Main results (Outcome)	Conclusions
Yao et al. [10] (2017) - China	To evaluate the effect of excess weight on lung function and FeNO in Asian children with a focus on changes in atopy	Smoking (Analysis was performed including and excluding individuals with asthma, but this was not an initial exclusion criterion)	Prospective cohort	1717 Asian children aged 5 to 18 years	Spirometry (Spiro Lab II, Medical International Research, Roma, Italy) FeNo (CLD 88 NO analyzer^®^, Evo Medics, Duernten, Switzerland)	Spirometry: ATS – position not mentioned FeNO: ATS and ERS - position not mentioned	FVC, FEV_1_, FEV_1_/FVC, PEF, FEF_25–75%_, FeNO	There were associations (+) of z-score of BMI with FVC, FEV_1_, PEF and FEF_25–75%_ and (−) with FEV_1_/FVC and FeNO. The associations occurred for the variables in the analysis with the entire group and excluding individuals with asthma	Excess weight changes lung volume and flow in a disproportionate manner, which reflects in the increase in FVC, FEV_1_, PEF and FEF_25–75%_, and reduction in FEV_1_/FVC
Peng et al. [11] (2016) - China	To evaluate whether weight index is associated with high BP, reduced FVC, dental caries and low vision, as well as whether nutritional status can predict diseases in schoolchildren	Yes (individuals with chronic or infectious diseases, e.g., cardiovascular, renal, hepatic, diarrhea, pneumonia, upper respiratory tract infection and influenza)	Cross-sectional	12,297 children aged 6 to 18 years	Spirometry (model not mentioned)	Standardized instrument not mentioned - standing	FVC	Group 6 to 12 years - males compared to females: higher weight, WC, BMI, SBP, FVC, low weight, overweight and obesity, FVC/weight; and lower SAH and low vision. Group 13 to 18 years - males: higher weight and height, WC, BMI, SBP, FVC, FVC/weight, prevalence of underweight, overweight, obesity, abdominal obesity and poor FVC/weight; and lower DBP, SAH, caries and low vision. Group of individuals with underweight had the lowest value of WC, SBP, FVC and visual acuity; and better FVC/weight. Group of individuals affected by obesity presented higher value of WC, SBP, DBP and FVC; and lower number of caries and FVC/weight. Children with overweight and obesity were at increased risk of high BP and poor FVC/weight when compared to children with normal weight, while undernourished children were at higher risk of caries, and in both groups, there was a higher risk of low vision	Inadequate nutritional status was associated with BP, FVC, caries and visual acuity. The prevalence of common diseases in school-aged children is greater in children with altered weight. Thus, weight index is a potential marker to predict some diseases, reinforcing the importance of maintaining weight in the prevention of diseases in schoolchildren. However, the causal relationship and physiological mechanisms to explain the changes need to be further studied
LoMauro et al. [12] (2016) - Italy	To verify whether the thoracoabdominal volume of male adolescents with obesity during exercise has specific characteristics to deal with the increasing ventilatory demand and to investigate whether a short period of multidisciplinary program for weight loss, including respiratory muscle resistance training, can modify the geometry and volume of the rib cage	Not mentioned	Prospective and intervention	11 male adolescents (Tanner 3 to 5), with standard deviation of BMI > 2, in relation to the Italian standards	Spirometry (MedGraphics CPX/D, Medical Graphics Corp., Saint Paul, Minn., USA) and OEP (Smart System BTS, Milan, Italy)	Spirometry: ERS – standing OEP: Standardized instrument and position not mentioned	FVC, FEV_1_, FEV_1_/FVC, PEF, total and compartmental volume in FRC and TLC, TV, RR, MV	FEV_1_/FVC was greater than 80% predicted in the individuals, indicating the absence of OVD, but with a possible indication of a restrictive pattern. After the intervention, there was an improvement in the absolute and predicted values of FVC, and a reduction in IC of the lung and abdominal rib cage was observed	Hyperinsulflation of the abdominal rib cage occurs during incremental exercise from moderate intensity to peak intensity in order to recruit lung volume, being an adaptation of the ventilatory dynamics to deal with the overload of the chest wall due to obesity, optimizing the synergism between the diaphragm and the abdominal musculature. The system starts to function at high volume to optimize lung compliance. After training, there was reduction in abdominal load, pulmonary recruitment and thoracic cavity volume, improvement of physical performance, reduction in dyspnea and delay in dynamic hyperinsulflation of the abdominal thoracic cavity without ventilatory and metabolic requirements, which contributes to the improvement of exercise tolerance and inhibition of the cycle of inactivity and weight gain
Özgen et al. [13] (2015) - Turkey	To evaluate the relation between lung function tests and functional capacity during exercise in children with obesity	Yes (syndromic children with endocrine conditions added to obesity, history or evidence of cardiovascular, respiratory or hepatic metabolic diseases)	Cross-sectional	74 children with obesity (13.4 ± 2.3 years) and 36 children without obesity (12.7 ± 1.9 years)	Spirometry (Spiro Lab III, MIR^®^, Rome, Italy)	ATS - position not mentioned	FVC, FEV_1_, FEV_1_/FVC, FEF_25–75%_, PEF	Lower FEV_1_, FEF_25–75%_ and distance covered in the 6MWT in the group with individuals affected by obesity. There was a (−) correlation of the distance covered in the 6MWT with the standard deviation of the BMI	Pulmonary and functional exercise capacity were lower among individuals with obesity
Kongkiattkul et al. [14] (2015) - Thailand	To evaluate the correlation between obesity indexes (anthropometry and bioimpedance) and lung function parameters and to identify whether the indexes correlate with abnormalities in lung function of children and adolescents with obesity	Yes (children with respiratory or neuromuscular diseases that could affect lung function assessment, respiratory infection within 2 weeks before the test, and inability to perform the tests)	Cross-sectional	45 individuals with obesity and aged 8 to 18 years	Spirometry and Body Plethysmography (Vmax 6200 Autobox™ diagnostic system - SensorMedics, Yorba Linda, CA, USA)	Standardized instrument and position not mentioned	FVC, FEV_1_, FEV_1_/FVC, FEF_25–75%_, FRC, TLC	64.4% of the individuals with obesity had a reduction in FRC, 7% had OVD (FEV_1_ < 80% and/or FEF_25–75%_ < 70% of predicted) and 2% RVD (TLC < 80% of predicted). There was a (−) correlation of FRC with BMI z-score, waist-height ratio, % of fat mass, fat mass index and % of fat in the trunk	FRC below normal was the most frequent alteration in lung function in the group of individuals affected by obesity. BMI z-score and obesity indexes that correlate with the central distribution of fat (waist-height ratio, % of fat mass, fat mass index and% of fat in the trunk) had a (−) correlation with FRC. The indices may help identify the reduction in FRC and should be used to assess obesity. The fat mass index > 17 kg/m^2^ may be a screening tool in obesity, risk for low FRC, and the need for respiratory care to prevent pulmonary complications
Ferreira et al. [15] (2014) - Brazil	To assess the influence of obesity on physical and lung function of children and adolescents with obesity and to associate the variables with a control group	Yes (chronic and/or respiratory diseases, neurological and/or physical limitations)	Cross-sectional	38 individuals with obesity and 39 healthy individuals aged 5 to 17 years	Spirometry (CPFS/D - MedGraphics Saint Paul, Minnesota, USA)	ATS and ERS - standing	FVC, FEV_1_, FEV_1_/FVC, FEF_25%_, FEF_50%_, FEF_75%_, FEF_25–75%_, PEF, ERV	The group of individuals affected by obesity showed lower FEV_1_/FVC, FEF_25%_, FEF_50%_, FEF_75%_, FEF_25–75%_, PEF, and distance covered in the 6MWT. Males were associated with lower FEF when compared to females in both groups	Changes in Spirometry associated with FEF alterations suggested obstructive change in 36.8% of individuals with obesity. The changes in lung function did not present a direct correlation with the performance in the 6MWT, but with the perception of effort in the exercise
Rastogi et al. [16] (2014) – United States of America	To investigate the association between total fat, trunk fat and metabolic abnormality with lung function of a sample of minority urban adolescents	Smoking [overweight individuals, chronic inflammatory conditions (rheumatologic, endocrine, gastrointestinal, or renal – study does not mention respiratory conditions)]	Cross-sectional	168 Hispanic and African adolescents aged 13 to 18 years (82 with obesity and 86 normal weight adolescents)	Spirometry and Body Plethysmography (SensorMedics, Yorba Linda, California)	ATS - position not mentioned	FVC, FEV_1_, FEV_1_/FVC, FEF_25–75%_, TLC, RV, RV/TLC, ERV, FRC, IC	Individuals had Spirometry levels within the parameters of normality. However, when they were characterized by BMI and WC, adolescents with total and trunk adiposity had lower % of predicted rates for pulmonary volumes, including RV, RV/TLC, ERV and FRC and higher IC. In the univariate analysis, adiposity and metabolic abnormality were predictors of lung function; high HOMA-IR predicts reduction in FEV_1_/FVC, RV, RV/TLC, ERV, FRC; and increased IC. Low HDL predicts low FEV_1_/FVC and RV/TLC; and high IC. Patients with asthma presented lower FEV_1_/FVC, without changes in lung volume. ERV was lower in males than in females. In the multivariate analysis adjusted for total fat and trunk, increasing HOMA-IR was a predictor of lower FEV_1_/FVC and ERV, and low HDL had a (+) correlation with FEV_1_/FVC. Total adiposity was predictor for IC, FRC, RV and RV/TLC; and adiposity in the trunk, RV and FRC. Among the covariates, asthma was a predictor for FEV_1_/FVC, females for ERV, and Hispanics for ERV and IC	Mechanical overload caused by adiposity and some metabolic abnormalities (HOMA-IR and HDL) were independent predictors of adolescent lung function deficit. The findings suggest that the early assessment of metabolic risk in children affected by obesity may help identify individuals at risk of developing lung diseases. However, studies are needed to understand the influence of the pathophysiology of obesity on lung function
Faria et al. [17] (2014) - Brazil	To investigate lung function response during exercise in adolescents with non-morbid obesity and without respiratory diseases	Yes (history of acute or chronic respiratory diseases, thoracic or skeletal deformity, heart diseases and congenital diseases)	Cross-sectional	92 adolescents aged 10 to 17 years – 47 with obesity (23 males) and 45 HC (21 males)	Spirometry (CPFS/D - MedGraphics Saint Paul, Minnesota, USA), FMR (Gerar^®^)	Spirometry: ATS and ERS - position not mentioned Manuvacuometry: Standardized instrument not mentioned - sitting	FVC, FEV_1_, FEV_1_/FVC, IC, ERV, VC MVV, MIP, MEP	Baseline BP and HR were higher among individuals with obesity, while SpO_2_ was lower. MVV, FVC and FEV_1_ were lower in males with obesity when compared to HC. IC in the group of females + obesity was higher than in the group of females + control. ERV was lower in both sexes among individuals with obesity when compared to controls. There were no differences in lung function before and after exercise. RMS showed differences between the sexes, but not between individuals with obesity and HC	The distribution of body fat alters lung function in a sex-dependent manner among individuals with obesity and does not change after exertion
Davidson et al. [18] (2014) - Canada	To investigate the relationship between age, sex and BMI and lung volume of healthy individuals aged 6 to 17 years	Yes (children with cardiorespiratory or ribcage diseases, asthma and individuals with reversible obstruction in spirometry)	Retrospective	327 healthy individuals divided into 4 groups: underweight (pBMI < 5), normal weight (pBMI between 5 and 85), overweight (pBMI between 85 to 95) and individuals with obesity (pBMI ≥95)	Spirometry, Body Plethysmography [SensorMedics (Northridge, CA) Vmax 22 system with volume measurement by the 6200 autobox and Vmax Legacy Plethysmography software (Viasys, Yorba Linda, CA)]	ATS - position not mentioned	FEV_1_, FVC, FEF_25–75%_, TLC, VC, FRC, ERV, RV, D_LCO_	Individuals with obesity showed lower values in the predicted % of FRC and ERV. RV was lower in the groups of individuals with overweight and obesity. Individuals with low weight had lower FVC and RV. In the group of individuals with obesity, there was lower FEV_1_/FVC. Additionally, there was a (+) linear association of the BMI z-score with the % of predicted of FVC, VC and D_LCO_ and (−) linear association of the BMI z-score with the % predicted of FRC, ERV, RV and absolute value of FEV_1_/FVC	Obesity was related to lower lung volume in children and adolescents. Changes in lung function may result in worsening respiratory symptoms and reduced functional capacity. Thus, there is a need to develop and implement effective strategies to prevent and manage obesity in childhood and adolescence
Gibson et al. [19] (2014) - Australia	To evaluate (i) whether children and adolescents with overweight or obesity can be submitted to submaximal exercise; (ii) respiratory limitations during exercise in children and adolescents with overweight and obesity compared to the control group	Yes (children with chronic cardiorespiratory problems or if it was not safe to exercise due to medical and/or musculoskeletal conditions)	Cross-sectional and prospective	26 individuals with obesity and 25 HC aged 10 to 18 years	Spirometry (SensorMedics, Yorba Linda, CA, USA), multi-breath nitrogen wash-out (Vmax 29, hardware and software - Sensormedics)	Spirometry: ATS - position not mentioned	FVC, FEV_1_, FEF_25–75%_, FRC, TLC, RV	There was no difference between the groups for TLC, FVC, FEV_1_ and FEF_25–75%_. However, the groups with individuals affected by overweight and obesity presented lower z-scores of the FRC and RV. The expiratory flow during the submaximal exercise was associated with the variables of weight (z-score BMI, weight and % of fat mass) and FEF_25–75%_	Young individuals with overweight and obesity may perform submaximal tests, and they tend to have a higher limitation of expiratory flow during submaximal exercise than healthy children. The use of compensatory breathing strategies allows overweight individuals to exercise at this intensity without feeling short of breath
Rio-Camacho et al. [20] (2013) - Spain	To investigate the left ventricular mass (echocardiography) of children with obesity, with and without metabolic syndrome; to evaluate the association between the level of adipokine and circulating cytokine and the alteration of left ventricular mass and Spirometry; and to determine the best variable to predict cardiovascular risk	Yes (children with chronic diseases and/or Tanner = 5 (to avoid sexual dysmorphism of adipokines analyzed at this stage)	Cross-sectional and descriptive	41 individuals with obesity and over 8 years old (20 with metabolic syndrome criterion)	Spirometry (Frow Screen - Jaegger^®^)	ATS - position not mentioned	FVC, FEV_1_, FEV_1_/FVC, FEF_25–75%_	MCP-1, LAR and CRP were higher in the presence of metabolic syndrome. There were no differences between the groups, with and without metabolic syndrome, for Spirometry and left ventricular mass	Obesity with metabolic syndrome has a higher degree of inflammation, and CRP is the best predictor of vascular risk. However, left ventricular mass and Spirometry were not influenced by the chronic inflammatory state in children and adolescents with obesity
Berntsen et al. [21] (2011) - Norway	To evaluate whether lung function measured in standing position is higher in children with overweight and obesity, when compared to the sitting position	The study does not mention respiratory conditions, only organic causes or diseases that may lead to obesity, conditions that may restrict the ability of being physically active and the use of medication that acts on growth or weight gain	Randomized and cross-over	115 individuals with overweight and 92 individuals with obesity aged 7 to 17 years	Spirometry (Vmax Series, SensorMedics, Yorba Linda, CA, USA)	ERS - sitting and standing	FVC, FEV_1_, FEF_50%_, FEV_1_/FVC, PEF	15% of the patients had asthma. Females, when compared to males, showed higher value of FEV_1_ and FVC. FEV_1_, FVC and FEF_50%_ were higher in Spirometry performed in the sitting position when compared to the evaluation in the standing position. In the linear regression analysis, the % of BMI, diagnosis of asthma, use of corticosteroids and sex were associated with changes in FVC, FEV_1_, FEF_50%_ and PEF	FVC, FEV_1_ and FEF_50%_ were higher in the sitting position when compared to the standing position. However, the increase had little clinical significance. In this way, the sitting position is the most appropriate posture to perform forced expiratory flow-volume maneuver
Chen et al. [22] (2009) - Canada	To evaluate WC as a predictor of lung function markers and to compare it with BMI in children and adolescents	Not mentioned (8 participants were excluded because they presented reduced lung function value and low reproducibility)	Cross-sectional	718 individuals aged 6 to 17 years	Spirometry (MedGrahics System CPFS - Medical Graphics Corporation, St. Paul, MN, 1992)	ATS - position not mentioned	FVC, FEV_1_, FEV_1_/FVC	WC had a (+) correlation with FVC and FEV_1_, and (−) correlation with FEV_1_/FVC. On average, 1 cm increase in WC was associated with an increase of 7 mL in FVC and 4 mL in FEV_1_. The assessment of height showed no changes in the association between WC and lung function. However, by adding body weight, the 1 cm increase in WC was associated with an increase of 4 mL in FVC and 2 mL in FEV_1_	The association (−) of WC and FEV_1_/FVC may be related to adiposity and/or lower predictability of FEV_1_ in relation to FEV_1_/FVC in children. Thus, overweight and obesity is likely to be associated with reduced lung function in childhood
Kalhoff et al. [23] (2011) - Germany	To investigate whether overweight or obesity are associated with abnormalities in IOS in a random sample of pre-school children aged 6 years	Not mentioned	Cross-sectional	518 preschoolers aged 6 years	IOS (MasterScreen IOS - CareFusion, Höchberg, Germany)	ATS and ERS - position not mentioned	Airway resistance at 5 Hz and pulmonary reactance at 5 Hz	The study found no differences in resistance and reactance at 5 Hz in children with high BMI	In children aged 6 years, abnormalities in IOS were not associated with increased BMI. IOS requires little cooperation to have the test performed, unlike Spirometry. Therefore, this technique enables the analysis of pulmonary development with the age by measurements in series, from childhood to adolescence
Gundogdu et al. [24] (2011) - Turkey	To evaluate the effects of obesity on lung function and to define the relation of BMI as independent variable and PEF as dependent	Yes (children who had major dysfunctions - cardiac, respiratory, renal or hematological or those with asthma symptoms)	Cross-sectional	1439 children aged 6 to 14 years	PFE (Mini Wright Peak Flow)	GINA, 2005 - standing	PEF	Simple multiple linear regressions showed reduced PEF associated with an increase in BMI category. PEF was lower in the group of individuals affected by obesity when compared to individuals without obesity	PEF was lower in children with obesity than in children with normal weight. As PEF is an indicator of pulmonary airflow resistance, there was an increase in respiratory resistance in children with obesity. The association of high BMI with reduced PEF indicated that obesity is a risk factor for reduced airflow and lung function. Reduced prevalence of asthma may be a result of the patients´ awareness and obesity prevention, i.e., prevention of obesity can reduce respiratory symptoms
Ferreira et al. [25] (2017) - Brazil	To evaluate lung function of children and adolescents with obesity (without asthma) by Spirometry and VC and to compare them to HC of the same age group	Yes (children with a history of respiratory diseases – asthma, obstructive sleep apnea or chronic obstructive pulmonary disease)	Cross-sectional	38 individuals with obesity and 39 HC aged 5 to 17 years	Spirometry (CPFS/D - Medical Graphics Corp., MN, USA and software Breeze PF 3.8 - Medical Graphics Corp., MN, USA) and VolC (CO2SMO - Dixtal, São Paulo, Brazil)	Spirometry: ATS and ERS - position not mentioned VolC: Standardized instrument not mentioned - sitting	FVC, FEV_1_, FEV_1_/FVC, FEF_75%_, FEF_25–75%_, ERV, MV, MV_alv_, TV, TV_alv_, DSV, DSV/TV, IC EtCO_2_, VCO_2_, RR, SpO_2_, Slp_2_, Slp_3_, Slp_2_/TV, Slp_3_/TV	The lowest z-score of FEV_1_/FVC, FEF_75%_ and FEF_25–75%_ occurred in the group of individuals with obesity, and 36.8% of individuals affected by obesity had FEF_25–75%_ lower than 70% (OVD by flow), and changes did not occur in the control group. In CV, the group of individuals with obesity showed lower DSV/TV and Slp_3_/TV. In the linear regression, the BMI z-score influenced FVC, FEV_1_/FVC, FEF_75%_, FEF_25–75%_, TV_alv_, DSV/TV, VCO_2_, Slp3 and Slp3/TV. There was no response to BD among individuals with obesity. In the division by age group (5 to 11 years or > 11 years) there were changes of FEV_1_/FVC in both groups and only in the older individuals for expiratory flow and pulmonary volume	Even without the diagnosis of asthma by clinical criteria and without response to BD, individuals with obesity show lower FEV_1_/FVC and FEF, indicating an obstructive process. In CV, in the group with individuals with obesity, there was higher TV_alv_, with no alteration in ventilation homogeneity, suggesting that these individuals have altered flow, but no changes in lung volumes
Del-Rio Navarro et al. [26] (2013) - Mexico	To compare bronchial hyperreactivity by the methacholine challenge testing in Mexican children with normal weight. In addition, to associate the group with normal weight with children with obesity or morbid obesity	Yes (underweight children, chronic respiratory diseases – including asthma and rhinitis, acute respiratory infection in the last month, endocrine diseases, dysmorphic dysfunction or exposure to tobacco)	Cross-sectional	229 children aged 10 to 18 years (40 – normal weight, 116 – with obesity and 73 – with morbidly obesity)	Spirometry (Vmax, Sensor Medics, Anaheim, CA), methacholine challenge testing (provocholine, 100 mg, Methaparm, Inc., Coral Springs, Fl) performed with dosimeter (Mark Salter Labs, Arvin, CA)	Spirometry: ATS - sitting	FVC, FEV_1_, FEF_25–75%_, PEF	In the group with obesity or morbid obesity, there was higher FVC and lower FEF_25–75%_, when compared to children with normal weight. Individuals with obesity, when compared with morbidly obese ones, had lower FEF_25–75%_. PEF was higher among children with obesity when compared to children with normal weight or with morbid obesity. During the methacholine challenge testing, FEV_1_ was lower among children with obesity than in children with morbid obesity, starting from a dose of 0.25 mg/mL up to a dose of 16 mg/mL. In the comparison of group of individuals with normal weight with the group of individuals with obesity, there was higher value of FEV_1_ during methacholine challenge testing with 0.25 and 1 mg/mL of methacholine and lower with 4 and 16 mg/mL in the control group	Obesity did not change aerobic responsiveness due to the use of methacholine and studies should be performed to confirm the findings
Spathopoulos et al. [27] (2009) - Greece	To evaluate the effect of obesity on lung function in a cohort of children aged 6 to 11 years and to associate obesity, atopy and asthma	Yes [high or low respiratory infection, exacerbation of asthma in the last 3 weeks, uncontrolled asthma (GINA), congenital heart abnormality, thoracic deformity or neuromuscular diseases]	Cohort	2715 children aged 6 to 11 years, (1978 – normal weight, 357 – with overweight and 300 – with obesity)	Spirometry (Vitalograth 2120)	ATS and ERS - position not mentioned	FVC, FEV_1_, FEV_1_/FVC, FEF_25–75%_	Among overweight individuals, FVC, FEV_1_, FEF_25–75%_ and FEV_1_/FVC levels were lower when compared to controls. Although the diagnosis of atopy and asthma is frequent in children with overweight and obesity, there was no difference in lung function in individuals with and without asthma. High BMI was an independent variable to predict reduction in Spirometry (mainly for FEF_25–75%_) and a risk factor of asthma and atopy. When separated by sex, high BMI was associated with FVC in females and FEV_1_/FVC in males	High BMI is a marker of obesity in children that can be easily measured and can determine the reduction in Spirometry measures, risk of atopy (both sexes) and asthma among females
Jeon et al. [28] (2009) – South Korea	To evaluate the factors that influence lung function in female adolescents, focusing on the hormonal factors of the menstrual cycle and obesity	Not mentioned	Cross-sectional	103 Korean high school children aged 15 to 18 years	Spirometry (Super Spiro, Micro Medical LTD, Kent, UK)	Standardized instrument and position not mentioned	FVC, FEV_1_, FEF_25–75%_, FEV_1_/FVC	FEV_1_/FVC was lower in females with obesity, when compared to HC of the same gender. The individuals who were evaluated in the menstrual period had lower FEV_1_, FEV_1_/FVC and FEF_25–75%_	The literature is scarce on the study of asthma, lung function and puberty. In the study, there was a limitation of airflow associated with obesity, allergy, menstrual cycle and sensitization by inhaled allergens. Studies should be conducted to evaluate the relationship between gender hormones, leptin, lung function and asthma
He et al. [29] (2009) - China	To evaluate the relationship of obesity and asthma, asthma symptoms and lung function of Chinese schoolchildren using the definition of overweight and obesity of a Chinese group	Not mentioned	Cross-sectional	2179 children (1138 boys and 1041 girls) aged 8 to 13 years	Spirometry (Minato AS-505 portable electric spirometer - Minato Ltd., Tokyo, Japan)	ATS - sitting	FVC, FEV_1_, FEF_25–75%_, FEF_75%_, FEF_25%_	2% of the sample had asthma. Overweight children had higher FVC than in HC. Men with overweight and women with obesity had higher FEV_1_ than controls	Lung function was not altered by obesity; however, there was a higher prevalence of respiratory symptoms in individuals with overweight or obesity. Longitudinal studies need to assess the cause-effect relationship between overweight, obesity and lung function
Silva et al. [30] (2011) - Brazil	To assess the onset of EIB in children and adolescents, without asthma and overweight	Yes (acute and chronic lung diseases, cardiopathy, diabetes, musculoskeletal deformity and pain, steroidal and non-steroidal anti-inflammatory medication, symptoms of viral infection (cold or flu) in the last 6 weeks and FEV_1_/FVC < 80%, FEV_1_ and PEF < 70% of predicted	Cross-sectional	69 school children aged 8 to 15 years (39 children with obesity without asthma and 30 HC without respiratory diseases)	Spirometry (EasyOne^®^ model 2001 - Zurich, Switzerland) and PFE (Peak flow meter Healthscan^®^ Personal Best)	ATS - position not mentioned	FEV_1_, PEF, FVC, FEV_1_/FVC, FEF_25–75%_	The prevalence of EIB was 62% in the group of individuals with obesity and 16% in the control group. There was no difference in Spirometry between groups, except for PEF, which was lower in the group of individuals with obesity	PFE was important in EIB diagnosis. Possibly, different etiologies are related to EIB and studies of pathophysiology of the central and peripheral airways and the onset of EIB in children and adolescents with excess weight should be performed
Bekkers et al. [31] (2013) – The Netherlands	To associate WC and BMI with lung function in 8-year-old children	Not mentioned	Cohort	1,106 children aged 7.4 to 9.2 years	Spirometry (Jaeger pneumotachograph - Viasys Healthcare, San Diego, CA)	ATS and ERS - sitting	FVC, FEV_1_, FEV_1_/FVC	Children with lower or higher WC showed lower FVC and FEV_1_ than those with normal WC. Children with low or high BMI had lower FVC and FEV_1_ when compared to normal BMI. Following the adjustments for confounding parameters, there were no differences between groups. Males with high BMI had lower FEV_1_/FVC when compared to the same sex with normal BMI. After adjustments for BMI, females with higher WC presented lower FEV_1_/FVC	In patients aged ~ 8 years, higher BMI or increased WC were not associated with FEV_1_ or FVC, demonstrating that this association may change over the course of childhood to adulthood
Assumpção et al. [32] (2017) - Brazil	To compare IOS parameters of children with normal weight, overweight and obesity	Yes (history of wheezing, respiratory diseases, respiratory tract infection in the last 2 weeks prior to assessment, muscle disorder, passive smoker, neurological diseases, asthma and/or allergic rhinitis [ISAAC - ≥ 5 (6 to 9 years) and 6 (10 to 14 years) for asthma and ≥ 4 (6 to 9 years) and 3 (10 to 14 years) for allergic rhinitis]. Visual, auditory or cognitive impairment, and individuals who did not understand the evaluation procedures	Cross-sectional, analytical and comparative	81 children aged 6 to 14 years: 30 – HC, 21 – with overweight and 30 – with obesity	IOS and Spirometry (IOS Jaeger™ - MasterScreen™ IOS, Erich Jaeger, Germany)	IOS: ATS - position not mentioned Spirometry: ATS and ERS - position not mentioned	Z5, R5, R20, X5, AX, Fres, FVC, FEV_1_, PEF, FEV_1_/FVC, FEF_25–75%_	Spirometry markers were within normal range and no differences between the 3 evaluated groups were determined. However, IOS in the group of individuals with obesity presented higher value than in the control group for: absolute value of Z5, and absolute value and % of predicted of R5, Fres and AX. The group with individuals with overweight presented higher value than the control group for % of predicted value of R5, Fres and AX and for absolute Fres	Children with obesity had higher IOS value, which represents obstruction of the airway in comparison with children with normal weight. Some changes occurred among children with overweight
Cibella et al. [33] (2015) - Italy	To investigate the effects of weight on lung function of healthy children in a sample registered in 2 cross-sectional surveys with selected age group	Yes (history of wheezing, night cough or cough due to exercise)	Cross-sectional	2,393 Caucasian individuals aged 10 to 17 years (51.1% boys)	Spirometry (Microloop, Miro Medica, Chatham Maritime, Kent, UK)	ATS and ERS - position not mentioned	FVC, FEV_1_, FEV_1_/FVC, FEF_25–75%_, FEF_25–75%_ /FVC	In the control of the variables weight, height, age and sex in the multiple linear regression, the weight B coefficient was (+) for FVC and FEV_1_, being higher for FVC, and (−) for FEV_1_/FVC and FEF_25–75%_/FVC. In the division by age group (< 11, 12, 13 and > 14 years), there was a (+) association of the B weight coefficient with FVC and FEV_1_ and a (−) association with FEV_1_/FVC and FEF_25–75%_ /FVC (association of FEF_25–75%_ and B weight coefficient did not occur in the group < 11 years). Among individuals with obesity and overweight, the % of predicted for FVC and FEV_1_ was higher and the absolute value of FEV_1_/FVC and FEF_25–75%_/FVC was lower than in HC	FVC and FEV_1_ were positively associated with weight, when corrected for height. However, due to a different magnitude in the effect of weight on FVC and FEV_1_, FEV_1_ showed a disproportionately smaller growth with weight gain when compared to FVC. Therefore, in individuals with a high BMI, there is a reduction of FEV_1_/FVC and FEF_25–75%_/FVC, and this change does not depend on respiratory symptoms
Silva et al. [34] (2015) - Brazil	To evaluate the effects of posture on thoracoabdominal kinematics of children with obesity and to compare them with a control group with normal weight	Yes (pulmonary or neuromuscular diseases)	Cross-sectional	35 children aged 8 to 12 years (18 with obesity and 17 with normal weight)	Spirometry (Micromedical Microloop MK8, Kent, England), RMS (digital manometer - MVD Globalmed 300, São Paulo, Brazil), OEP (OEP - BTS Bioengineering, Italy)	Spirometry: ATS and ERS - sitting; maximum respiratory pressure - sitting; OEP: Aliverti and Pedotti, 2003 - sitting and supine	FVC, FEV_1_, FEV_1_/FVC, MEP, MIP, TV variation, VTRCp, VTRCa, VTAB, VTRCp%, VTRCa%, VTAB%, Ti, Te, MV, RR, TV and θ	MIP, MV and TV were higher among individuals with obesity. The posture influenced TV (total and compartmental). There was higher TV, VTRCp and VTRCa in the sitting position, while VTAB was higher in supine position among children with obesity. TV was more influenced by the compartments VTRCp% and VTRCa% in the sitting position, while VTAB% was higher in the supine position. In addition, VTAB% was higher among individuals with obesity	The study demonstrated that the thoracoabdominal kinematics of children with obesity is influenced by the supine position, with an increase in abdominal contribution and reduction in the contribution of the rib cage to ventilation, suggesting that supine areas of pulmonary hypoventilation may occur. However, the thoracoabdominal kinematics was not different in the sitting position between the groups. Sitting posture is recommended during therapeutic procedures to achieve better distribution of regional rib cage volume and pulmonary ventilation
Torun et al. [35] (2014) - Turkey	To compare lung function in children with normal weight, overweight, obesity or morbid obesity and to evaluate the effects of degree of obesity on lung function	Yes (atopy or chronic lung diseases, asthma or family history of asthma, atopic dermatitis, food intolerance or syndrome)	Cross-sectional	170 individuals (30 – with overweight, 34 – with obesity, 64 – with morbid obesity and 42 – with normal weight) aged 9 to 17 years	Spirometry (MIR, Spirolab III colour, Roma, Italy)	Standardized instrument and position not mentioned	FVC, FEV_1_, FEV_1_/FVC, FEF_25–75%_, PEF	Overweight, obesity and morbid obesity showed lower FEF_25–75%_ and PEF, when compared to the group of individuals with normal weight	The study considered FEV_1_/FVC < 80% of predicted as OVD. Thus, despite the difference, the study did not identify an obstructive abnormality in the group of individuals with obesity or morbid obesity individuals, when compared to controls with normal weight individuals and pointed out that longitudinal studies should investigate the effect of obesity degree and weight loss on lung function among individuals with obesity
Khan et al. [36] (2014) - Canada	To associate anthropometric measures and lung function in children	Not mentioned	Cross-sectional	1583 children aged 6 to 17 years (males: 573 – with normal weight, 216 – with obesity; females: 626 – with normal weight and 168 – with obesity)	Spirometry (Koko)	ATS and ERS - sitting	FVC, FEV_1_, FEV_1_/FVC, FEV_0,75_	There was higher FVC, FEV_0.75_ and FEV_1_ in males than in females, and the opposite occurred in FEV_1_/FVC. When the variable was adjusted according to the sex of the participants, there was association of BMI and WC with residual FVC in males and FVC and residual FEV_1_ in females. Both sexes had an inverse correlation of BMI with residual FEV_1_/FVC. In the division by body mass, in the individuals with normal weight, there was a (+) effect of the BMI on FVC, FEV_0.75_ and FEV_1_, and a (−) effect on FEV_1_/FVC. WHR had a (+) correlation with FVC and FEV_1_ and a (−) correlation with FEV_1_/FVC. The WHR presented a (−) correlation with FEV_1_/FVC. In children with overweight and obesity, there was a (−) association of WC and WHR with FVC and FEV_1_. In this group, there was a (−) correlation of the skinfold of the triceps, biceps, iliac crest and medial calf with FVC, FEV_0.75_ and FEV_1_, and the same was observed for the subscapular fold and the sum of all folds, adding the association with FEV_1_/FVC. In HC, there was a correlation between: (i) triceps skinfold and FVC; (ii) iliac crest fold and FEV_1_/FVC; (iii) sum of the 5 folds (triceps, biceps, subscapular, iliac crest and medial calf) and FEV_1_/FVC	In males, there was worsening of lung function with overweight. Lung function was altered by abdominal and subcutaneous fat, and skinfolds were more sensitive to measure adiposity when compared to anthropometric data. The best indicator of adiposity in the analysis of lung function in males was the triceps skinfold
Rosa et al. [37] (2014) - Brazil	To evaluate RMS by maximum respiratory pressure in healthy, schoolchildren with overweight and obesity, and to identify whether the anthropometric and respiratory variables are related to the outcomes	Yes (ISAAC)	Cross-sectional	90 school children aged 7 to 9 years (30 – with obesity, 30 – with overweight and 30 – HC	Spirometry (PIKo-1, Spire Health, USA) and RMS (one-way valve digital manovacuometer (MVD 300, G-MED, Brazil)	Spirometry: ATS/ERS - sitting FMR: ATS - sitting	FEV_1_, MIP, MEP	There was higher MIP in HC when compared to the others. The correlation of age with FEV_1_, MIP and MEP was (+), and of MIP with BMI (−). MIP and MEP correlated with each other and, with less intensity, with the FEV_1_. MEP had a (+) correlation with height and FEV_1_. In the individual analysis of the groups, there was correlation of age with weight and height, except in the group of individuals with overweight; weight with height and BMI; MIP with age in the HC group, FEV_1_ in the group of individuals with obesity and MEP in the 3 groups; MEP with age and height in the HC group and FEV_1_ in the 3 groups; FEV_1_ with age in the 3 groups, and weight and height in the HC group and in the group of individuals with overweight	Obesity and overweight were associated with lower MIP when compared to HC. There was a correlation between MIP and MEP with age and FEV_1_, mainly, obesity. MIP correlated with BMI and MEP with height, mainly in HC. Thus, possibly, the anthropometric variables may influence RMS in children, as well as in the relation between strength and FEV_1_
Assunção et al. [38] (2014) - Brazil	To describe pulmonary functional alterations in asymptomatic and overweight children and adolescents	Yes (history of wheezing, cough, chest pain, or known lung diseases)	Cross-sectional and descriptive	59 individuals aged 8 to 18 years (4 – with overweight, 28 – with obesity and 27 – with morbid obesity)	Spirometry (Koko Digidoser - Ferraris Respiratory, Louisville, CO, USA) and helium washout (mass flow sensor Vmax 21) (Viasys Healthcare, Palm Springs, CA, USA)	Spirometry: ATS and Brazilian Society of Pulmonology and Phthisiology - position not mentioned helium washout - Standardized instrument and position not mentioned	FVC, FEV_1_, RV, TLC, FEV_1_/FVC, FEF_25–75%_	30.3% of individuals had TLC < 80% of predicted and 3.5% TLC > 120%. In the sample, 25.5% of the individuals had a (+) response to BD in FEV_1_, most of them with morbid obesity. Individuals with (+) response to BD had FEV_1_/FVC < than LLN, therefore, OVD. Regarding the use of BD and FVC, 2 individuals had a (+) response. Other findings were: 32.2% of individuals with OVD (15.2% - with overweight or obesity and 16.9% - morbid obesity) 25.4% with RVD (11.8% - with overweight or obesity and 13.5% - morbid obesity), and 6.7% with MVD (3.3% - with overweight or obesity and 3.3% - with morbid obesity). In addition, there was a (−) correlation between BMI with WC and FEV_1_/FVC in the MVD group	Asymptomatic respiratory individuals with excess weight had a high prevalence of ventilatory disorders, predominantly OVD. Additionally, there was a (+) response to the BD, higher than that reported in the literature, most frequently in morbid obesity
Van de Griendt et al. [39] (2012) – The Netherlands	To evaluate the effects of weight reduction on lung function in children with morbid obesity in children aged 8 to 18 years	Yes (asthma or regular use of inhaled corticosteroids)	Longitudinal	112 children aged 8 to 18 and with BMI ≥ 30 Kg/m^2^ with comorbidities or BMI ≥ 35 Kg/m^2^	Spirometry and Body Plethysmography (MasterScreen PFT + body box - Jaeger Viasys, Wuerzburg, Germany)	Standardized instrument and position not mentioned	FEV_1_, FEF_50%_, ERV, FRC, TLC and FuncVC	After 6 months of treatment to reduce weight, there was an increase of 3.08% in FuncVC, 2.91% in FEV_1_, 2.27% in TLC and 14.8% in ERV. WC had a (−) correlation with ERV. Changes in BMI score correlated with ERV	Weight reduction in children with morbid obesity may improve lung function, especially for ERV and airflow limitation
Alghadir et al. [40] (2012) – Saudi Arabia	To investigate the relationship between severity of obesity and parameters of lung function, comparing lung function in Saudi men with overweight and obesity with individuals with normal weight, and to compare the value found with the reference values for Caucasian individuals	Not mentioned (an interview and questionnaire about the medical history of lung infection were conducted, but did not mention this factor as exclusion criterion)	Cross-sectional	60 male individuals aged 6 to 13 years (20 in each group: with obesity, with overweight and with normal weight)	Spirometry (Pony FX - COSMED, Italy	Spirometry: ATS – sitting	FEV_1_, FVC, FEV_1_/FVC	The more fat a child has, the more compromised the lung function will be. Saudi children had lower value than predicted for height and age. In the group of individuals with obesity and overweight, there was a lower (predicted) value for FVC and FEV_1_ and higher for FEV_1_/FVC. Lung function of children with obesity was lower than that of the other groups, and the difference between the % of the measured value and the % of the predicted showed higher value in obesity for FVC and FEV_1_ than in HC or individuals with overweight	Lung function of male Saudi Arabians with obesity or overweight was lower than that of children of the same age range in the HC group. The difference in relation to the predicted for their ages may indicate restriction in thoracic expansion and affect exercise capacity
Paralikar et al. [41] (2012) - India	To evaluate lung function in adolescents with obesity in the city of Baroda, Gujarat	Yes (cough)	Cross-sectional	60 male individuals aged 12 to 17 years (30 – with obesity and 30 – HC)	Spirometry (MEDI: SPIRO - Maestros Mediline Systems Ltd., Navi Mumbai, India)	Spirometry: ATS and ERS - sitting	FEV_1_, FVC, FEV_1_/FVC, PEF, FEF_25–75%_, MVV	The mean % of predicted FEV_1_ was lower in the group of individuals affected by obesity, as well as the mean absolute value and % of predicted FEV_1_/FVC and MVV values. However, no individuals had OVD. Weight, BMI and WC had a (−) correlation with FEV_1_/FVC, MVV and FEF_25–75%_, and WHR with MVV and FEF_25–75%_	Lung function among individuals with obesity was lower than that of the HC, being obesity a health risk in the evaluated age group. Despite the difference between groups, no individual had OVD or RDV. Longitudinal studies are needed to understand the relationship between increased body weight and lung function
Supriyatno et al. [42] (2010) - Indonesia	To determine the prevalence of abnormalities in lung function among Indonesian male adolescents and young people with obesity	Yes (children with exacerbated asthma)	Cross-sectional	110 children with obesity aged 10 to 12 years	Spirometry (PS7 Spirometer)	Spirometry: Polgar, 1971 - position not mentioned	FVC, FEV_1_, FEV_1_/FVC, FEF_25%_, FEF_50%_	In the sample, there was history of 29.1% asthma, 41.8% allergic rhinitis, 58.2% abnormality in lung function (30% MVD (obstructive and restrictive), 25.5% RVD and 2.7% OVD]	Abnormalities in lung function occur in obesity in early adolescence, with the most frequent change being MVD. There was no correlation between BMI and lung function. Studies are needed to assess the association of the degree of obesity and abnormalities in lung function with more accurate measures to assess body fat and with HC

*FeNo* Fraction of exhaled nitric oxide, *ATS* American Thoracic Society, *ERS* European Respiratory Society, *FVC* Forced vital capacity, *FEV*_*1*_ Forced expiratory volume in the first second of forced vital capacity, *FEV*_*1*_*/FVC* Relation between forced expiratory volume in the first second and forced vital capacity, *PEF* Peak expiratory flow measured by spirometry, *FEF*_*25–75%*_ Forced expiratory flow between 25 and 75% of forced vital capacity, *BMI* Body mass index (weight/height^2^), *HC* Healthy controls, *BP* Blood pressure, *WC* Waist circumference, *SBP* Systolic Blood Pressure, *FVC/weight* Forced vital capacity index by weight, *Tanner* Pubertal developmental stage according to Tanner’s criteria, *OEP* Optoelectronic plethysmography, *FRC* Functional residual capacity, *TLC* Total lung capacity, *TV* Tidal volume, *RR* Respiratory rate, *MV* Minute volume, *6MWT* Six-minute walk test, *FEF*_*25%*_ Forced expiratory flow at 25% of forced vital capacity, *FEF*_*50%*_ Forced expiratory flow at 50% of forced vital capacity, *FEF*_*75%*_ Forced expiratory flow at 75% of forced vital capacity, *ERV* Expiratory reserve volume, *RV* Residual volume, *RV/TLC* Ratio of residual volume and total lung capacity, *IC* Inspiratory capacity, *HOMA-IR* Homeostasis model assessment of insulin resistance, *HDL* High density lipoprotein, *MVV* Maximum voluntary ventilation, *MIP* Maximal inspiratory pressure, *MEP* Maximum expiratory pressure, *WHR* Waist hip ratio, *HR* Heart rate, *SpO*_*2*_ Peripheral oxygen saturation, *pBMI*, BMI percentile, *D*_*LCO*_ Diffusing capacity of the lungs for carbon monoxide, *VC* Vital capacity, *MCP-1* Monocyte Chemotactic Protein-1, *LAR* Leptin to adiponectin ratio, *CRP* C-reactive protein, *IOS* Impulse oscillometry, *VolC* Volumetric capnography, *tot* Total, *alv* Alveolar, *DSV* Dead space volume, *DSV/TV* Relation between Dead space volume and tidal volume, *Slp*_*2*_ Slope of phase 2, *Slp*_*3*_ Slope of phase 3, *Slp*_*2*_*/TV* Relation between slope of phase 2 and tidal volume, *Slp3/TV* Relation between slope of phase 3 and tidal volume, *EtCO*_*2*_ End-tidal carbon dioxide, *VCO*_*2*_ Volume of exhaled carbon dioxide, *CI* Capnography index [(Slp_2_/Slp_3_)× 100], *Hz* Hertz, *EIB* Exercise-induced bronchospasm, *Z5* Respiratory impedance, *R5* Total resistance, *R20* Central airway resistance, *X5* Reactance at 5 Hz, *AX* Reactance area, *Fres* Resonant frequency, *VTRCp* Tidal volume of the pulmonary rib cage, *VTRCa* Tidal volume of the abdominal rib cage, *VTAB* Tidal volume of the abdomen, *VTRCp%* Percentage of contribution of the tidal volume in the rib cage to total tidal volume, *VTRCa%* Percentage of contribution of the tidal volume in the abdominal rib cage to the total tidal volume, *VTAB%* Percentage of abdominal tidal volume contribution to tidal volume, *Ti* Inspiratory time, *Te* Expiratory time, *θ* Phase transition between 2 compartments, *FuncVC* Functional vital capacity, *LLN* Lower limit of normal, *OVD* Obstructive ventilatory disorder, *RVD* Restrictive ventilatory disorder, *MVD* Mixed ventilatory disorder, *FEV*_*0.75*_ Forced expiratory volume at 0.75 s, *SAH* Systemic arterial hypertension, *DBP* Diastolic blood pressure, *BD* Bronchodilator, *PFE* Peak expiratory flow measured by Peak Flow Meter, *GINA* Global Initiative for Asthma

## Results

As described in the methods, in brief, the articles were selected in three stages. A total of 48 articles were selected. After the exclusion of duplicates, 33 articles were included in the systematic review.

Table 2 shows a detailed informative and descriptive summary of the articles in this review: authorship, year of publication, place of study (country), study objective, presence or absence of respiratory disease, type of study, type of evaluation of lung function and type of posture and markers used in the analysis, main results and conclusions.

A wide age range (5 to 18 years) could be observed in the studies, with a higher prevalence between 11 and 13 years, which was an inclusion criterion in 69.7% (23/33) of the articles [11, 13–15, 17–22, 24–27, 29, 30, 32, 33, 35, 36, 38–40].

It is important to highlight the exclusion of studies that included participants with respiratory diseases, since the focus was to evaluate the pulmonary changes resulting exclusively (or with the least possible influence of other factors) from obesity. Therefore, including children with previous respiratory diseases could evolve into sampling (selection of individuals), confounding (proven impact on outcomes) or information bias (previous knowledge of an existing problem). In this context, 66.7% (22/33) [12–15, 17–21, 25, 26, 29, 32–37, 39–42] of the studies excluded individuals with respiratory diseases, 27.3% (9/33) [16, 22–24, 27, 28, 30, 31, 38] did not mention respiratory diseases as a factor of exclusion or non-inclusion, and 6% (2/33) [11, 43] excluded only individuals with a history of smoking. Among the studies that excluded previous respiratory diseases, several exclusion criteria could be observed: some authors excluded only individuals with exacerbation of asthma or cough; others excluded any respiratory conditions that might impair the evaluation; and others used standardized instruments such as the ISAAC questionnaire (The International Study of Asthma and Allergies in Childhood). This demonstrates the variability of methods adopted by the authors of the different studies, low standardization of exclusion/inclusion criteria, and difficulties/limitations to evaluate, diagnose and exclude patients with possible respiratory diseases in some specific cases among the evaluated children and adolescents [44].

Additionally, in the studies analyzed, the inclusion of healthy controls (HC), without obesity, was described as a criterion to compare lung function. In this context, 78.8% (26/33) [11, 13, 14, 16–20, 24–38, 41–43] of the studies compared individuals affected by obesity with HC.

The articles included were produced in 18 countries, with a predominance of European (8) [21–23, 31, 34, 35, 40], South American (8) [17, 18, 26, 32, 33, 37, 39, 41] and Asian (7) [11, 13, 15, 27, 30, 31, 42] countries. Also, 4 studies from North America were included [19, 24, 38, 43], as well as one from Central America [29], one from Oceania [20] and four from intercontinental countries ( three Euroasians [14, 25, 36] and one from Asia and Oceania [12]).

In the evaluation of lifestyle habits, 15.2% (5/33) [20, 24, 30, 41, 42] of the studies assessed the participation of individuals in physical activities. Also, 9.1% (3/33) [16, 20, 42] of the studies assessed screen time of the participants and one [24] study mentioned the use of a lifestyle habits questionnaire but did not detail the assessed variables.

There was no uniform definition of obesity among studies: 27.3% (9/33) [11, 14, 15, 20, 35–37, 40, 42] used references by Cole et al. [45–47]; 21.2% (7/33) [12, 17, 19, 26, 29, 38, 43] used the criteria established by the Center for Disease Control and Prevention (CDC); 15.1% (5/33) [13, 18, 33, 39, 41] used the definition of the World Health Organization (WHO); 12.1% (4/33) [16, 24, 32, 34] did not mention any references for the definition of obesity; and 27.3% (9/33) [14, 21–23, 25, 27, 28, 31, 36] used references according to their countries of origin. Two [14, 36] of the studies mentioned above, used references by Cole et al. [45–47] in addition to references according to their countries of origin.

Inflammatory markers were assessed and correlated with lung function in just 6% (2/33) [11, 21] of the studies, which is a quite low percentage, considering the systemic and complex nature of obesity, which requires an interdisciplinary approach. Among the studies that assessed inflammatory process, one study evaluated fraction of exhaled nitric oxide (FeNO) [11] and another serum levels of C-reactive protein (CRP), adiponectin, leptin, interleukin 6 (IL-6), tumor necrosis factor (TNF-α), monocyte chemoattractant protein-1 (MCP-1), visfatin and retinol binding protein 4 [21].

Spirometry was the most commonly used tool to assess lung function, i.e., in 93.9% (31/33) [11–24, 26, 27, 29–43] of the studies. Next, body plethysmography and measurement of the respiratory muscle strength (RMS) were the most used tools in 12.1% (4/33) [15, 19, 40, 43] and 9.1% (3/33) [18, 37, 41] of the studies, respectively. Optoelectronic plethysmography (OEP) [23, 37], impulse oscillometry (IOS) [28, 33] and peak expiratory flow measured by peak flow meter (PFE) were included in the analyses of 6.1% (2/33) [25, 32] of the studies. Other tools were used in only one study each: nitrogen [20] and helium [39] washout, FeNO [11], volumetric capnography (VolC) [26] and methacholine challenge testing [29].

Among the spirometry variables, forced expiratory volume in the first second (FEV_1_) of the forced vital capacity (FVC) was the most prevalent marker in the studies, included in 90.9% (30/33) [11, 12, 14–24, 26, 27, 29–43] of the analyses, followed by FVC and the FEV_1_/FVC ratio, used in 87.9% (29/33) [11–24, 26, 27, 29–39, 42, 43] and 72.7% (24/33) [11, 12, 14–18, 21–24, 26, 27, 31–39, 42, 43] of the studies, respectively. Forced expiratory flow between 25 and 75% of FVC (FEF_25–75%_) was also a widely assessed marker, being analyzed in 57.6% (19/33) [11, 14, 15, 17–21, 29–36, 39, 42, 43] of the studies. In addition, PEF and PFE were included in 33.3% (11/33) [11, 14, 17, 22, 23, 25, 29, 32, 36, 42] of the studies.

Among the variables analyzed using other tools besides spirometry, total lung capacity (TLC) and FRC could be observed in 21.2% (7/33) [15, 19, 20, 23, 39, 40, 43] and 18.2% (6/33) [15, 19, 20, 23, 40, 43] of the studies, respectively.

The comparison of the studies with a control group that showed comparative values or significant associations is described in Table 3. In this context, a great number of studies for each variable could be observed due to the selection of the various markers evaluated. No clear pattern emerged, as regards FEV_1_ and FVC, with about half of the studies reporting no association between obesity and lung function parameters [11, 14, 16–20, 26, 27, 29–38, 41–43]. A clearer pattern emerged as regards FEV_1_/FVC and FEF_25–75%_, as most studies found either a negative association or no association between obesity and lung function [11, 14, 16–20, 26, 27, 29–38, 41–43], with only one study reporting a positive association (Table 3) [13].

**Table 3:** Descriptive analysis of the markers evaluated in lung function in individuals aged 5 to 18 years

Variable	Association			
	+	–	No difference	Total
FVC	8	5	10	23
FEV_1_	4	6	13	23
FEV_1_/FVC	1	10	7	18
FEF_25–75%_	1	7	8	16
Peak expiratory flow measured by spirometry	2	4	3	9
Expiratory reserve volume	–	3	2	5
Functional residual capacity	–	3	–	3
Total lung capacity	–	–	3	3
Residual volume	–	3	–	3
Inspiratory capacity	2	–	–	2

+, studies that showed comparative markers with greater value in obesity or positive associations with variables that are indicative of obesity; −, studies that showed comparative markers with lower value in obesity or with negative association with variables that are indicative of obesity; FVC, forced vital capacity; FEV_1_, forced expiratory volume in the first second of forced vital capacity; FEV_1_/FVC, forced expiratory volume in the first second and forced vital capacity ratio; FEF_25–75%_, forced expiratory flow between 25 and 75% of forced vital capacity

## Discussion

### Effects of growth and development on lung function

Childhood and adolescence are characterized by major changes in the structure and functions of the human body systems. The physiological processes that influence lung function in 6-year-old children are different from those influencing 15-year-old adolescents, even if we disregard other multiple factors, such as gender, ethnicity, environment and genetics. Changes in lung function of adults with obesity are related to increased intra-abdominal pressure due to the deposition of fat in this region, which compromises the efficiency of diaphragmatic mobility, as well as the deposition of fat on the rib cage, which reduces its compliance. However, such aspects are insufficient to understand the influence of obesity on lung function of children and adolescents [48, 49].

**Fig. 3:**
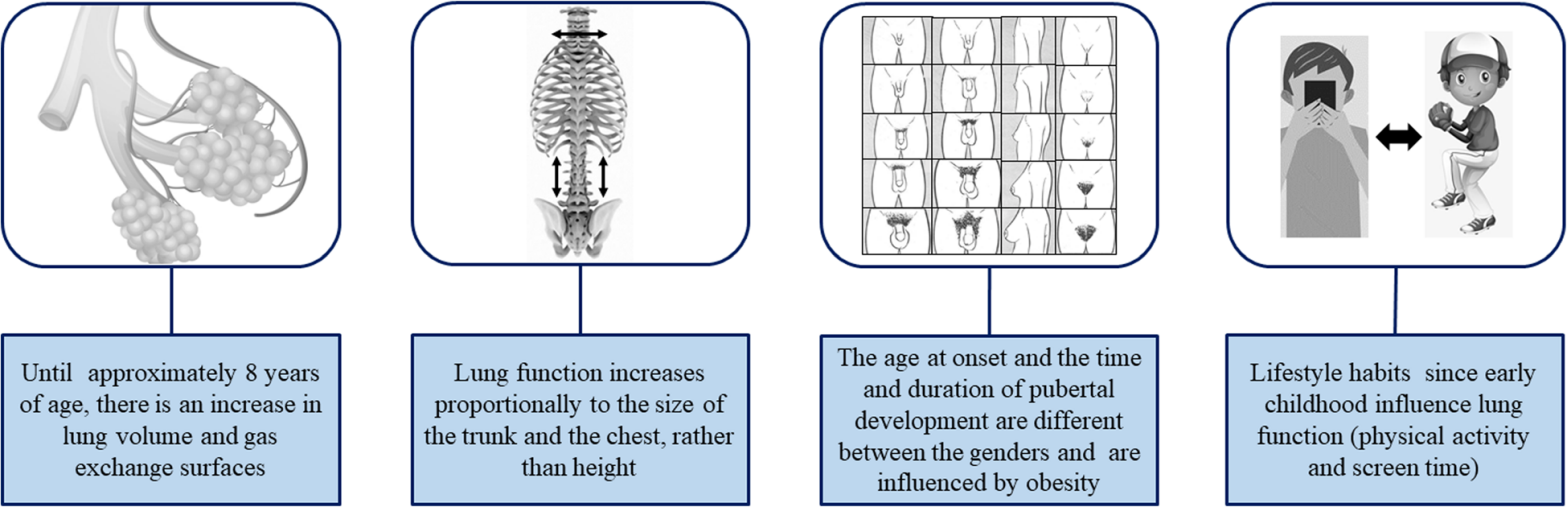
Factors that influence lung function in children and adolescents. The images used in the figure are not under copyright

Figure 3 lists some factors that influence lung function in children and adolescents and that should be considered in the discussion about the variability of the findings in this systematic review. The first factor is the increase in lung volume and surface for gas exchange until approximately eight years of age. In this context, 45.5% (14/33) [11, 13, 16, 17, 22, 24–28, 33, 34, 38, 41] of the studies in this review included individuals under 8 years old – a period characterized by airway growth and development. Among these studies, 13 [11, 13, 16, 17, 22, 24–27, 33, 34, 38, 41] included, at the same time, individuals over eight years of age – a period, in which the respiratory tract is anatomically formed.

According to the literature, until the end of the preschool age, the respiratory tract growth follows a dysanaptic pattern, i.e., the growth of the airways is slower than the growth of the lung parenchyma. After this period, the growth is isometric, showing greater homogeneity. This pattern of growth up to early childhood may lead to increased airway resistance and higher risk of obstructive processes in these individuals, especially in males, who have proportionally smaller airways than females during this maturation period. Thus, the findings regarding lung function in children and adolescents should be analyzed considering the lung growth phase and the child’s development [49, 50].

The second and third factors described in Fig. 3 are interrelated and associated with changes arising from growth. The onset of puberty marks the beginning of a process of maturation, characterized by body and psychological changes. The changes vary according to gender, and in women, the pubertal development begins approximately two years earlier than in men. Moreover, hormonal changes may directly influence lung function: at first, by growth spurts, followed by increase in trunk height and ribcage diameter, which influence the increase in lung capacity and volumes. Another example is the increase in the production of male testosterone during puberty, which triggers a muscle growth peak, which includes the respiratory muscles and favors the increase of FVC and respiratory flows [51, 52].

Among individuals affected by obesity, the changes mentioned in the previous paragraph tend to occur at an earlier stage. Although the mechanisms are not yet well-established, studies have shown a relationship between insulin resistance and increased serum levels of leptin. Thus, comparing individuals of the same age group, at different stages of pubertal development and different genders, may be a bias in the analysis of lung function. Of the studies included in the systematic review, 21.2% (7/33) [14, 16, 21, 23, 32, 38, 43] analyzed pubertal development [53, 54].

The last item described in Fig. 3 is fundamental to understand lung function in children and adolescents and is associated with physical activities and sedentary lifestyle habits. In this context, we should emphasize that the comprehensive knowledge of the studied sample is of utmost importance. Today, the majority of the population is sedentary, and sedentary behaviors are not exclusively associated to the group of individuals affected by obesity. So, if the sample is composed of individuals with obesity under treatment/follow-up and HC with sedentary lifestyle habits, there may be a bias in relation to cardiorespiratory conditioning, which may affect lung function. Therefore, in the analysis of lung function in children and adolescents with obesity, information about physical activities and screen time is fundamental [55, 56].

Interestingly, despite the importance of this data, only 15.1% (5/33) [20, 24, 30, 41, 42] of the studies in this systematic review assessed participation in physical activities, 9.1% (3/33) [16, 20, 42] included the assessment of screen time, and 1 [24] study mentioned lifestyle habits without detailing the assessment items used.

### Measurement tools to define obesity

Body mass index (BMI) was the most commonly used tool to determine obesity in children and adolescents. However, the criteria to define obesity varied among the studies. The option of using country-specific standards of normality or those published by the CDC or WHO, determined the lack of homogenization of the samples. Also, the cut-off points for the definition of obesity within the selected references were different. Therefore, the use of comparable criteria between the studies would allow a more precise definition regarding the presence of obesity in children and adolescents. Comprehensive references, including data collection at a global level, are best indicated as they analyze normality patterns, taking ethnic differences into account.

The amounts of body fat and lean mass make the assessment of lung function in individuals with obesity more reliable. This can be explained because BMI, which is the most commonly used indicative of obesity, shows some limitations. BMI measures excess weight rather than excess fat and the variability due to gender, age, ethnicity and lifestyle habits may act as modifiers. Thus, well-trained individuals with high lean mass indexes are classified with obesity [57, 58].

In this systematic review, only 12.1% (4/33) [15, 18, 23, 38] of the studies used instruments that allowed the quantification of body fat. The first study used bioimpedance (BIA) [23], which estimates fat mass, fat-free mass and total body water. The second study [18] used BIA and the skinfold measurement test, which estimates the amounts of fat in each segment. However, the literature reports that this method has its limitations for the assessment of individuals with obesity [59]. The third study [38] only analyzed the skinfolds, and the fourth [15] used BIA and dual energy X-ray absorptiometry (DXA), which is a more accurate tool, as DXA assesses the amount and distribution of fat and lean body mass. The analysis of the distribution of body fat is an important tool to detect alterations caused by impaired respiratory mechanics, which is greater with the increase in fat in the thorax and abdomen.

### Obesity epidemic reflected on the diversity of the studied populations

The inclusion of studies with individuals from almost all continents, except for Africa, is relevant for the analysis of the findings. They reflect a global epidemic of obesity among children and adolescents. According to the WHO, the prevalence of overweight and obesity among children aged 5 to 19 years increased from 4% in 1975 to 18% in 2016. Currently, more than 124 million children and adolescents are affected by excess of weight. Weight gain trends include both developed and underdeveloped countries, and currently, overweight and obesity are more prevalent and more often associated with causes of death than underweight, except in some parts of Africa (especially sub-Saharan Africa) and Asia [60, 61].

### Trends related to lung function in children and adolescents with obesity

The variability of the results showed an inability to establish lung function changes in children and adolescents with obesity. However, some trends have been detected and are discussed as follows.

Among the variables analyzed, the comparison of FEV_1_/FVC between individuals with obesity and HC was observed in 54.5% (18/33) [11, 12, 14, 16–18, 26, 27, 31–38, 42, 43] of the studies and of those, 55.6% (10/18) [11, 12, 16, 17, 26, 31, 34, 35, 38, 42] found lower value or negative association of the variable with obesity. The described changes may be an indication of the obstructive disorder in individuals affected by obesity during childhood and adolescence. The obstruction is related to pro-inflammatory activity of the adipose tissue, which could trigger bronchial hyperreactivity. Prior to our study, a review described similar findings regarding FEV_1_/FVC [62].

The analysis of inflammatory markers to identify systemic impacts caused by obesity was reported in only 6% (2/33) [11, 21] of the studies. One study [21] did not make any references to comparisons with the HC group. But, in another study [11], a positive association of FeNO with BMI was determined, suggesting that inflammation was more often detected in individuals affected by obesity and that these inflammatory changes should be considered in the clinical evaluation.

Despite the changes in FEV_1_/FVC, in the 22 [11, 14, 16–20, 26, 27, 29–38, 41–43] studies that analyzed FEV_1_ in children and adolescents with obesity and HC, there were discrepancies in the findings to confirm the presence of obstructive ventilatory disorder: (i) 56.5% (13/23) [17, 19, 20, 26, 29, 31–34, 36, 37, 41, 43] found no differences or associations between the groups; (ii) 26.1% (6/23) [14, 16, 18, 27, 38, 42] found lower value or a negative association between FEV_1_ and obesity; (iii) 17.4% (4/23) [11, 24, 30, 35] found higher value or a positive association in individuals with obesity. Thus, FEV_1_ was not associated with lung function impairment in overweight individuals.

FEF_25–75%_ should also be considered, as it is a marker of obstructive ventilatory disorder. Some studies indicate that this tool is more sensitive than FEV_1_, and it can detect early ventilatory changes, especially in the small airways [63, 64]. However, as with most variables, there was also variability in the results. In total, 48.5% (16/33) [11, 14, 17, 19, 20, 26, 29–36, 42, 43] of the studies analyzed FEF_25–75%_ of individuals with obesity and HC and of those: (i) 43.8% (7/16) [19, 20, 30–33, 43] found no differences and associations between indicators of obesity and FEF_25–75%_; (ii) 46.7% (8/16) [14, 17, 26, 29, 34–36, 42] found lower value or a negative association of FEF_25–75%_ with obesity; (iii) one (6.6%) [13] found a positive association between FEF_25–75%_ and obesity in children and adolescents.

In short, FEV_1_/FVC was the spirometry marker with the greatest sensitivity to identify a possible obstructive process due to obesity in children and adolescents. However, the variability of this marker – and, even more of other indications of obstruction – was high, regardless of the study. Thus, the development of cohort studies on indicatives of growth stages and body development is of utmost importance, since these factors are different between individuals affected by obesity and HC and also influence lung function. Pubertal development or age cohorts considering the time of dysanaptic growth and isometric growth would considerably reduce confounding factors and allow better understanding of lung function changes due to obesity in children and adolescents.

In the evaluation of FVC, which indicates restrictive respiratory disorder, 66.7% (23/33) [11, 13, 14, 16–20, 24, 26, 27, 29–38, 42, 43] of the studies included comparison with HC and 43.5% (10/23) [14, 17, 20, 31–33, 36, 37, 42, 43] of them did not find any differences between individuals with or without obesity. Only 21.7% (5/23) [16, 18, 27, 34, 38] found a negative association or lower values of FVC in children and adolescents with obesity. In a systematic review conducted in 2012, the authors concluded that the literature references demonstrated an association between reduced FVC and FEV_1_ with obesity in children and adolescents, in disagreement with our findings [65].

Besides the spirometry variables, some other measures contributed to the analysis of lung function in children and adolescents with obesity. TLC, FRC and residual volume (RV) were markers used in only 9.1% (3/33) [19, 20, 43] of the studies comparing individuals with obesity and HC. All these studies reported that individuals affected by obesity showed lower value or a negative association of FRC and RV with obesity. However, there were no differences in relation to TLC. If TLC – which is the sum of the inspiratory capacity (IC), and FRC (FRC = RV + ERV) – does not present a difference between individuals with obesity and HC, and if FRC and RV are reduced, it can be assumed that IC should be higher in individuals affected by obesity. Only 2 [18, 43] studies analyzed this variable and found a higher value or a positive association of IC with obesity.

These results are in agreement with a systematic review published in 2016, which evaluated the effects of obesity on lung volume and capacity in children and adolescents, and found a reduction in some markers in obesity, especially the reduction in FRC, ERV and RV [66].

### Issues on respiratory physiology and biomechanics requiring further investigation

The values for respiratory mechanics of individuals with obesity are incoherent. Some hypothesis and questions can be raised, namely:

(i). Are individuals with obesity actually “stronger” and are, therefore, able to inspire more air?
(ii). However, if there is an increase in RMS, considering the 9 [11, 14, 17, 25, 29, 32, 33, 36, 42] studies that analyzed peak expiratory flow measured by spirometry (individuals with obesity x HC), why did only 2 [11, 29] studies find a positive association with obesity? Why did 4 [17, 25, 32, 36] studies find lower value or a negative association between obesity and peak expiratory flow measured by spirometry? And, why did 3 [14, 33, 42] studies find no differences between groups?
(iii). Are lung function changes in individuals with obesity due to the differences between males and females, since in females, there is a pattern of gynoid obesity, with higher fat concentration on the hip and legs; whereas in males, an android pattern occurs, with more volume of fat in the chest and abdomen?
(iv). It is well-known that individuals affected by obesity tend to initiate pubertal development earlier than healthy individuals. So, is there greater muscle development among individuals with obesity, which tends to be balanced at the end of puberty? Can impairment of lung function in individuals with obesity be clearly observed after puberty?

### Confounding biases to clarify the mechanisms that interfere with lung functions in children and adolescents affected by obesity

Obesity is a multisystemic dysfunction, and therefore it is difficult to control the variables in order to understand the damage caused to lung function. For this reason, we found high variability in the results. Given the studies included in this systematic review, we are not able to establish which ventilatory changes are due to obesity in children and adolescents, even excluding data whose focus was the influence of asthma on obesity, which is a bias in this analysis. There are mechanisms that correlate both dysfunctions and the causal relationship between them may hinder perception of what is actually a consequence of obesity and/or asthma [8–10].

The findings of this review, although inconclusive, may give us a direction for future research. The strategies include: greater sampling control; reduction of confounding variables; conducting interdisciplinary and longitudinal studies with individuals with obesity versus HC; detailed analysis of environmental and social aspects; validation of findings among different populations; larger sample size; inclusion of measurements of lean mass and fat mass in order to unify and establish better criteria to define obesity; and future studies aiming to associate different genetic aspects, with predisposition to variability for weight gain, as well as for the individual nuance of lung function.

Thus, the inclusion and analysis of lung function in children and adolescents have become fundamental. Pubertal staging should be considered in order to avoid the influence of early maturation of individuals affected by obesity on the overestimation of lung capacity. It is important to analyze fat distribution, considering the concentration of abdominal and thoracic fat as factors that directly influence lung function. For this analysis, the use of instruments, such as DXA, may help determine the influence of the distribution and amount of body fat on lung function.

Assessing RMS with manuvacuometry or the distribution of lean mass with DXA, or even amount of lean mass using BIA, also favours the understanding of the physical conditions that influence lung function in children and adolescents. In order to analyze physical conditions, it is also important to include the evaluation of programmed and non-programmed physical activities as well as the screen time.

It is essential to exclude previous respiratory conditions that may influence lung function and control variables that are indicatives of inflammation, including CRP, erythrocyte sedimentation rate, FeNO or serum levels of leptin, adiponectin, IL-6 and TNF-α, which will allow precise determination of the influence of overweight in lung function of children and adolescents with obesity.

### Meta-analysis

Meta-analysis is the gold standard in order to interpret a specific topic such as the importance of lung function in cases of obesity in the pediatric population. However, as described in our data there is no standardization in the studies about lung function in children and adolescents with obesity. To perform a meta-analysis, a minimum of standardization should be applied in the data acquisition. However, looking for the data included in Table 2, the studies were performed using different methodologies and/or lung function tools and/or lung functions measures (markers). Moreover, the age range was not equal, and the objectives were different among the studies. In brief, articles [11, 13–17, 19, 20, 22, 25, 26, 28–30, 32–38, 40–42] were described as cross-sectional studies on children-adolescents without respiratory diseases, where spirometry markers (such as FVC, FEV_1_ and FEV_1_/FVC) were assessed, and the relation between lung function and body mass, expressed as either BMI or norm weight/overweight/obesity, was estimated. Those studies did not allow us to perform a meta-analysis because there is a disparity of study objectives, population type (age range, sex distribution), origin of the population, presence of other lung function measurement, obesity as an independent variable and the exercise analysis. The information about the disparities among the studies is shown in Table 2 and Table 4.

**Table 4:** Disparities between the markers of cross-sectional studies on children-adolescents without respiratory diseases, where spirometry markers were assessed, and the relation between lung function and body mass, expressed as either body mass index or norm weight/overweight/obesity preventing the performance of meta-analysis

Authors (year)	Objective	Spirometry measurement						Other lung function measurment	Obesity as an independent variable	Exercise
		FVC	FEV_1_	FEV_1_/FVC	FEF_25–75%_	PEF	ERV			
Peng et al. [11] (2016) - China	To evaluate whether weight index is associated with high blood pressure, reduced FVC, dental caries and low vision, as well as whether nutritional status can predict diseases in schoolchildren	Yes	No	No	No	No	No	–	No	No
Özgen et al. [13] (2015) - Turkey	To evaluate the relation between lung function tests and functional capacity during exercise in children with obesity	Yes	Yes	Yes	Yes	Yes	No	–	Yes	Yes
Kongkiattkul et al. [14] (2015) - Thailand	To evaluate the correlation between obesity indexes (anthropometry and bioimpedance) and lung function parameters and to identify whether the indexes correlate with abnormalities in lung function of children and adolescents with obesity	Yes	Yes	Yes	Yes	No	No	+ Body Plethysmography	Yes	No
Ferreira et al. [15] (2014) - Brazil	To assess the influence of obesity on physical and lung function of children and adolescents with obesity and to associate the variables with a control group	Yes	Yes	Yes	Yes	Yes	Yes	–	Yes	Yes
Rastogi et al. [16] (2014) – United States of America	To investigate the association between total fat, trunk fat and metabolic abnormality with lung function of a sample of minority urban adolescents	Yes	Yes	Yes	Yes	No	Yes	+ Body Plethysmography	No	
Faria et al. [17] (2014) - Brazil	To investigate lung function response during exercise in adolescents with non-morbid obesity and without respiratory diseases	Yes	Yes	Yes	Yes	No	Yes	+ Respiraory Muscular Force	Yes	Yes
Rio-Camacho et al. [20] (2013) - Spain	To investigate the left ventricular mass (echocardiography) of children with obesity, with and without metabolic syndrome; to evaluate the association between the level of adipokine and circulating cytokine and the alteration of left ventricular mass and spirometry; and to determine the best variable to predict cardiovascular risk	Yes	Yes	Yes	Yes	No	No	–	Yes	No
Gibson et al. [19] (2014) - Australia	To evaluate (i) whether children and adolescents with overweight or obesity can be submitted to submaximal exercise; (ii) respiratory limitations during exercise in children and adolescents with overweight and obesity compared to the control group	Yes	Yes	No	Yes	No	No	+ Multi-breath Nitrogen Wash Out	Yes	Yes
Chen et al. [22] (2009) - Canada	To evaluate waist circunference as a predictor of lung function markers and to compare it with BMI in children and adolescents	Yes	Yes	Yes	No	No	No	–	No	No
Ferreira et al. [25] (2017) - Brazil	To evaluate lung function of children and adolescents with obesity (without asthma) by Spirometry and volumetric capnography and to compare them to healthy control of the same age group	Yes	Yes	Yes	Yes	No	Yes	+ Volumetric Capnography	Yes	No
Del-Rio Navarro et al. [26] (2013) - Mexico	To compare bronchial hyperreactivity by the methacholine challenge testing in Mexican children with normal weight. In addition, to associate the group with normal weight with children with obesity or morbid obesity	Yes	Yes	No	Yes	Yes	No	+ Methacoline Challenge Testing	Yes	No
Jeon et al. [28] (2009) – South Korea	To evaluate the factors that influence lung function in female adolescents, focusing on the hormonal factors of the menstrual cycle and obesity	Yes	Yes	Yes	Yes	No	No	–	No	No
He et al. [29] (2009) - China	To evaluate the relationship of obesity and asthma, symptom of asthma and lung function of Chinese schoolchildren using the definition of overweight and obesity of a Chinese group	Yes	Yes	No	Yes	No	No	–	No	No
Silva et al. [30] (2011) - Brazil	To assess the onset of exercise-induced bronchospasm in children and adolescents, without asthma and overweight	Yes	Yes	Yes	Yes	Yes	No	+ Peak Expiratory Flow Meter	Yes	Yes
Assumpção et al. [32] (2017) - Brazil	To compare IOS parameters of children with normal weight, overweight and obesity	Yes	Yes	Yes	Yes	Yes	No	+ Impulse Oscillometry	Yes	No
Cibella et al. [33] (2015) - Italy	To investigate the effects of weight on lung function of healthy children in a sample registered in 2 cross-sectional surveys with selected age group	Yes	Yes	Yes	Yes	No	No	–	No	No
Silva et al. [34] (2015) - Brazil	To evaluate the effects of posture on thoracoabdominal kinematics of children with obesity and to compare them with a control group with normal weight	Yes	Yes	Yes	No	No	No	+ Respiraory Muscular Force + Optoelectronic Plethysmography	Yes	No
Torun et al. [35] (2014) - Turkey	To compare lung function in children with normal weight, overweight, obesity or morbid obesity and to evaluate the effects of degree of obesity on lung function	Yes	Yes	Yes	Yes	Yes	No	–	Yes	No
Khan et al. [36] (2014) - Canada	To associate anthropometric measures and lung function in children	Yes	Yes	Yes	No	No	No	–	Yes	No
Rosa et al. [37] (2014) - Brazil	To evaluate respiratory muscular strength by maximum respiratory pressure in healthy, schoolchildren with overweight and obesity, and to identify if the anthropometric and respiratory variables are related to the outcomes	No	Yes	No	No	No	No	+ Respiraory Muscular Force	Yes	No
Assunção et al. [38] (2014) - Brazil	To describe pulmonary functional alterations in asymptomatic and overweight children and adolescents	Yes	Yes	Yes	Yes	No	No	+ Helium Washout	Yes	No
Alghadir et al. [40] (2012) - Saudi Arabia	To investigate the relationship between severity of obesity and parameters of lung function, comparing lung function in Saudi men with overweight and obesity with individuals with normal weight, and to compare the value found with the reference values for Caucasian individuals	Yes	Yes	Yes	No	No	No	–	Yes	No
Paralikar et al. [41] (2012) - India	To evaluate lung function in adolescents with obesity in the city of Baroda, Gujarat	Yes	Yes	Yes	Yes	Yes	No	–	Yes	No
Supriyatno et al. [42] (2010) - Indonesia	To determine the prevalence of abnormalities in lung function among Indonesian male adolescents and young people with obesity	Yes	Yes	Yes	No	No	No	–	No	No

*ERV* Expiratory reserve volume, *FVC* Forced vital capacity, *FEV*_*1*_ Forced expiratory volume in the first second of forced vital capacity, *FEV*_*1*_*/FVC* forced expiratory volume in the first second and forced vital capacity ratio, *FEF*_*25–75%*_ Forced expiratory flow between 25 and 75% of forced vital capacity, *PEF* Peak expiratory flow measured by spirometry, *+* Studies that showed comparative markers with greater value in obesity or positive associations with variables that are indicative of obesity, − Studies that showed comparative markers with lower value in obesity or with negative association with variables that are indicative of obesity

## Conclusion

The different results observed for lung function in children and adolescents with obesity show that there is no consensus on the impairment in such individuals in the literature. Considering the influence of growth and development on the function of all systems, it is fundamental to control the variables to reduce sampling, information and confounding biases, as well as to enable the analysis of the deleterious effects of obesity. In this context, new studies should require greater control of variables that influence growth and development to better understand the influence of obesity on lung function of children and adolescents.

However, studies on individuals with obesity describe a trend towards lower FEV_1_/FVC, FRC, ERV and RV, suggesting that both mechanical and inflammatory impairments influence lung function throughout childhood and adolescence.

Studies on pubertal development would be significant for a standard comparison including hormonal and structural changes in this period and the onset and duration of maturation. The quantification and distribution of body fat and the analysis of lifestyle habits would promote coherence and standardization on this subject, favoring the clinical approach to individuals.

The prevalence of obesity has increased worldwide, and although it is a relevant public health problem that affects all age groups, the role and methods to evaluate its impact on lung function in children and adolescents have not been established yet, and full understanding of the topic is still far from being attained.

## Data Availability

This is a systematic review and we can give all the information about the articles used to develop the study if requested by a reader. MSF, FALM or JDR should be contacted to request the data.

## Abbreviations

-: Studies that showed comparative markers with lower value in obesity or with negative association with variables that are indicative of obesity
+: Studies that showed comparative markers with greater value in obesity or positive associations with variables that are indicative of obesity
6MWT: Six-minute walk test
alv: Alveolar
ATS: American Thoracic Society
AX: Reactance area
BD: Bronchodilator
BIA: Bioimpedance
BMI: Body mass index (Weight/Height^2^)
BP: Blood pressure
CDC: Center for Disease Control and Prevention
CI: Capnography index [(Slp_2_/Slp_3_)× 100]
CRP: C-reactive protein
DBP: Diastolic blood pressure
D_LCO_: Diffusing capacity of the lungs for carbon monoxide
DSV: Dead space volume
DSV/TV: Relation between dead space volume and tidal volume
DXA: Dual energy X-ray absorptiometry
EIB: Exercise-induced bronchospasm
Embase: Excerpta Medica Database
ERS: European Respiratory Society
ERV: Expiratory reserve volume
EtCO_2_: End-tidal carbon dioxide
FEF_25–75%_: Forced expiratory flow between 25 and 75% of forced vital capacity
FEF_25%_: Forced expiratory flow at 25% of forced vital capacity
FEF_50%_: Forced expiratory flow at 50% of forced vital capacity
FEF_75%_: Forced expiratory flow at 75% of forced vital capacity
FeNO: Fraction of exhaled nitric oxide
FEV_0.75_: Forced expiratory volume at 0.75 s
FEV_1_: Forced expiratory flow in the first second of forced vital capacity
FEV_1_/FVC: Relation between forced expiratory volume in the first second and forced vital capacity
FRC: Functional residual capacity
Fres: Resonant frequency
FuncVC: Functional vital capacity
FVC: Forced vital capacity
FVC/weight: Forced vital capacity index by weight
GINA: Global Initiative for Asthma
HC: Healthy controls
HDL: High density lipoprotein
HOMA-IR: Homeostasis model assessment of insulin resistance
HR: Heart rate
Hz: Hertz
IC: Inspiratory capacity
IL-6: Interleukin 6
IOS: Impulse oscillometry
ISAAC: The International Study of Asthma and Allergies in Childhood
LAR: Leptin to adiponectin ratio
LLN: Lower limit of normal
MCP-1: Monocyte chemotactic protein-1
MEDLINE: PubMed, Medical Literature Analysis and Retrieval System Online - Public Medline
MEP: Maximum expiratory pressure
MIP: Maximal inspiratory pressure
MV: Minute volume
MVD: Mixed ventilatory disorder
MVV: Maximum voluntary ventilation
OEP: Optoelectronic plethysmography
OVD: Obstructive ventilatory disorder
pBMI: BMI percentile
PEF: Peak expiratory flow measured by spirometry
PFE: Peak expiratory flow measured by Peak Flow Meter
R20: Central airway resistance
R5: Total resistance
RMS: Respiratory muscle strength
RR: Respiratory rate
RV: Residual volume
RV/TLC: Ratio of residual volume and total lung capacity
RVD: Restrictive ventilatory disorder
SAH: Systemic arterial hypertension
SBP: Systolic blood pressure
Slp_2_: Slope of phase 2
Slp_2_/TV: Relation between slope of phase 2 and tidal volume
Slp_3_: Slope of phase 3
Slp3/TV: Relation between slope of phase 3 and tidal volume
SpO_2_: Peripheral oxygen saturation
Tanner: Pubertal developmental stage according to Tanner’s criteria
Te: Expiratory time
Ti: Inspiratory time
TLC: Total lung capacity
TNF-α: Tumor necrosis factor
tot: Total
TV: Tidal volume
VC: Vital capacity
VCO_2_: Volume of exhaled carbon dioxide
VHL: Virtual Health Library (Brazil)
VolC: Volumetric capnography
VTAB: Tidal volume of the abdomen
VTRCa: Tidal volume of the abdominal rib cage
VTRCa%: Percentage of contribution of the tidal volume in the abdominal rib cage to the total tidal volume
VTAB%: Percentage of abdominal tidal volume contribution to tidal volume
VTRCp: Tidal volume of the pulmonary rib cage
VTRCp%: Percentage of contribution of the tidal volume in the rib cage to total tidal volume
WC: Waist circumference
WHO: World Health Organization
WHR: Waist hip ratio
X5: Reactance at 5 Hz
Z5: Respiratory impedance
θ: Phase transition between two compartments
